# Signatures of kin selection in a natural population of the bacteria *Bacillus subtilis*

**DOI:** 10.1093/evlett/qrad029

**Published:** 2023-07-18

**Authors:** Laurence J Belcher, Anna E Dewar, Chunhui Hao, Melanie Ghoul, Stuart A West

**Affiliations:** Department of Biology, University of Oxford, Oxford, United Kingdom; Department of Biology, University of Oxford, Oxford, United Kingdom; Department of Biology, University of Oxford, Oxford, United Kingdom; Department of Biology, University of Oxford, Oxford, United Kingdom; Department of Biology, University of Oxford, Oxford, United Kingdom

**Keywords:** cooperation, kin selection, public goods, population genetics, relatedness, inclusive fitness

## Abstract

Laboratory experiments have suggested that bacteria perform a range of cooperative behaviors, which are favored because they are directed toward relatives (kin selection). However, there is a lack of evidence for cooperation and kin selection in natural bacterial populations. Molecular population genetics offers a promising method to study natural populations because the theory predicts that kin selection will lead to relaxed selection, which will result in increased polymorphism and divergence at cooperative genes. Examining a natural population of *Bacillus subtilis*, we found consistent evidence that putatively cooperative traits have higher polymorphism and greater divergence than putatively private traits expressed at the same rate. In addition, we were able to eliminate alternative explanations for these patterns and found more deleterious mutations in genes controlling putatively cooperative traits. Overall, our results suggest that cooperation is favored by kin selection, with an average relatedness of *r* = .79 between interacting individuals.

## Introduction

Laboratory studies have suggested that bacteria cooperate in a diversity of ways (Strassmann et al., 2011; West et al., 2006). Individual cells produce and secrete molecules to collectively scavenge nutrients, fight antibiotics, and move through their environment (Dugatkin et al., 2005; Griffin et al., 2004; McNally et al., 2014). This cooperation is however vulnerable to cheating by nonproducers, which withhold their own cooperation while benefiting from that of others (Ghoul et al., 2014). A resolution to this vulnerability is kin selection, where cooperation is favored because the benefits of cooperation go to related cells that share the gene for cooperation (Hamilton, 1964). Laboratory experiments have also supported a role of kin selection, with experimental evolution, and by showing how clonal growth makes neighboring cells highly related, and limited diffusion keeps secreted molecules in the neighborhood (Diggle et al., 2007; Griffin et al., 2004; Kümmerli et al., 2009; Strassmann et al., 2000; Velicer et al., 2000).

In contrast, there is little evidence for cooperation and kin selection in natural populations of bacteria outside the lab, with the exception of *Pseudomonas aeruginosa* (Andersen et al., 2015; Butaite et al., 2017; Cordero et al., 2012; Ghoul et al., 2017). The extent to which bacteria cooperate and interact with close relatives is likely to be highly dependent on environmental conditions. It is hard to know whether the artificial environments and gene knockouts of lab experiments are representative of natural populations. Experiments have also shown that some traits can be cooperative in some environments but private in others (Jautzus et al., 2022). Across species, comparative studies have shown that cooperation is more common in species where relatedness is higher (Fisher et al., 2013; Simonet & McNally, 2021), but this does not help us determine whether specific traits are evolving as cooperative public goods. For this, we need a way to study bacteria in their natural environment.

**Figure 1.**
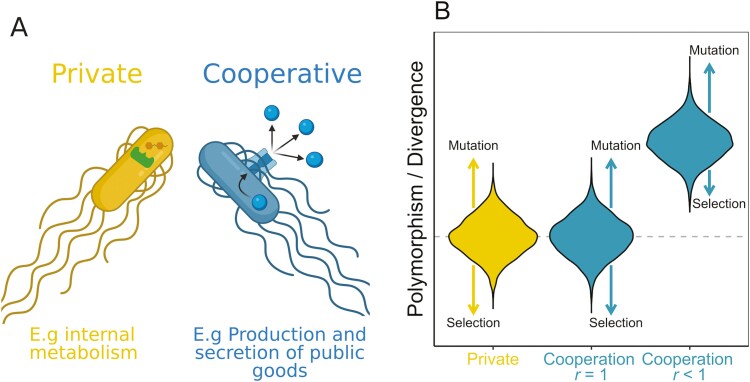
Population genetic theory for cooperative traits. (A) Representation of the categorization of traits as private (yellow) or cooperative (blue). Cooperative traits involve the production and secretion of molecules whose fitness benefits are shared with other nearby cells. Private traits are those whose fitness benefits are only felt by the individuals expressing the trait (B) Prediction for relative polymorphism and divergence for private (yellow) and cooperative (blue) genes. If relatedness, *r* = 1, then cooperative genes (middle blue violin) should have the same polymorphism and divergence as private genes (left yellow violin). In contrast, if *r* < 1, then cooperative genes (right blue violin) should show greater polymorphism and divergence than private genes. Figure based on Linksvayer and Wade (2009), van Dyken & Wade (2010) .

A combination of bioinformatics and molecular population genetics offers a promising method to test for cooperation and kin selection in natural populations. Theory from population genetics predicts that kin selection leaves a distinct signature (footprint) of selection, which we can detect in genome sequence data (Figure 1) (Linksvayer & Wade, 2009, 2016; Van Dyken & Wade, 2010, 2012; Van Dyken et al., 2011). Genes that code for private traits provide a direct benefit to the individual expressing them. Genes that code for cooperative traits provide indirect benefits to other cells in the population. In a nonclonal population, the cells that benefit from cooperation might not carry the gene for cooperation, as relatedness *r* < 1. If the benefits of cooperation are going to cells that do not carry the gene, then this relaxes selection on cooperative traits relative to private traits. In a clonal population, the cells that benefit from cooperation will also carry the cooperative gene, as relatedness *r* = 1. This means that selection will not be relaxed on cooperative traits in clonal populations, but will be relaxed in nonclonal populations.

The relaxed selection when *r* < 1 results in an increased probability of fixation for deleterious mutations and a decreased probability of fixation for beneficial mutations (14–16). The consequence of this change in fixation probabilities, when *r* < 1, is that it would lead to increased polymorphism and divergence in cooperative genes relative to genes that have direct fitness effects (Figure 1). Consequently, by examining patterns of polymorphism and divergence, we can test for signatures of cooperation favored by kin selection. This method was first applied to the signal and response QS genes across several species of bacteria (Van Dyken & Wade, 2012) and has since been applied to the social amoeba *Dictyostelium discoideum* and the bacteria *P. aeruginosa*. The results from *D. discoideum* were mixed, although relatedness is close to *r* = 1 in this species, so we might not expect a significant signature of kin selection (de Oliveira et al., 2019; Gilbert et al., 2007; Noh et al., 2018; Ostrowski et al., 2015). The results from *P. aeruginosa* found evidence for cooperative traits favored by kin selection (Belcher et al., 2022).

However, these previous studies were unable to account for some potentially confounding factors. First, the genomes had been collected in a variety of geographic locations and/or over a long period time. For example, the *P. aeruginosa* genomes were sampled over several decades and from six different continents (Belcher et al., 2022). The underlying population genetic theory assumes that all genomes are sampled from a single population, at a single time point. Violating this assumption could have led to biased or spurious results (Hahn, 2018). Second, previous analyses were unable to directly test whether genes have conditional fitness effects. If a gene only has fitness effects in certain environments or certain generations, then this can also lead to relaxed selection, with increased polymorphism and divergence (Van Dyken & Wade, 2010). For example, the study on bacteria compared to genes that may vary substantially in sociality and conditional expression (van Dyken & Wade, 2012), and the study on *P. aeruginosa* lacked data on gene expression, and so made targeted comparisons between cooperative and private traits that were likely to be co-expressed at the same time (Belcher et al., 2022). If this assumption did not hold, then their patterns could alternatively be explained by conditional expression rather than kin selection for cooperation (Van Dyken & Wade, 2010).

We were able to address these problems by taking advantage of two recent data sets in the bacteria *Bacillus subtilis*. *Bacillus subtilis* is found in soil and the gastrointestinal tracts of several animals, including humans, and is used on an industrial scale by biotechnology companies (Logan & De Vos, 2015). A number of laboratory studies have suggested that *B. subtilis* is a highly cooperative species, which secretes a number of potentially cooperative enzymes (Arnaouteli et al., 2021; Dragoš et al., 2018; Kalamara et al., 2018; Veening et al., 2008). First, we used a natural population consisting of 31 environmental isolates collected as part of a citizen science project in Dundee, Scotland (Kalamara et al., 2021) (Supplementary Table 1). All these strains were collected from the wild around the same time and from similar niches. Second, we were able to directly test our assumptions about genes being co-expressed at the same time, by using two genome-wide studies of gene expression across several time points during biofilm formation (Futo et al., 2021; Pisithkul et al., 2019). This gene expression data allowed us to test whether the groups of private and cooperative genes that we compare in our main analysis tend to be expressed together. An additional advantage of this population genetics approach is that it tests for signatures of kin selection over recent evolutionary time, and so provides an answer averaged across the different environments encountered in nature, rather than examining a single environment.

## Materials and methods

### Strains

We use the whole-genome sequences of 31 strains of *B. subtilis* (Kalamara et al., 2021). The strains are environmental isolates, collected from a citizen science project in Dundee where people brought soil samples from their garden. *Bacillus subtilis* is most commonly found in soil, but is also found living as a commensal in animal intestines (Tam et al., 2006) and in marine environments (Fan et al., 2011). While durable spores than can disperse through the air can allow long-distance migration (Roberts & Cohan, 1995), and strains do not phylogenetically cluster based on environment (Brito et al., 2018), several factors are in favor of using these strains as our natural population. First, these samples were all collected at the same time for the same project (Kalamara et al., 2021). Furthermore, we know that rates of migration between populations scales with geographic distance, most of the sequence diversity within the species is contained in local population (Roberts & Cohan, 1995), and there is evidence for fine-scale genetic structure in micro-scale populations (Duncan et al., 1994).

We downloaded raw sequence data for each strain from the European Nucleotide Archive (accession number PRJEB43128). The full list of strains can be found in Supplementary Table S1.

### Genes regulated by quorum sensing

For our set of quorum sensing (QS)-controlled genes, we combine three published data sets: First, 88 genes controlled by ComXAP (Comella & Grossman, 2005); second, 114 genes controlled by *degU* (Kobayashi, 2007); third, 40 genes controlled by Spo0A (Molle et al., 2003). We did not use a fourth possible data set, of 166 late competence genes affected by the transcription factor ComK, which are indirectly regulated by ComA (Berka et al., 2002). This is partly because our undomesticated reference strain (NCIB 3610) carries a plasmid-encoded protein that interferes with the competence machinery (Konkol et al., 2013) (Supplementary S7). In addition, we wanted to focus on the QS systems known to produce public goods (Figure 3 of Kalamara et al., 2018).

### Identifying social genes

We used an artisan approach to identify social genes, whereby we categorize genes as social based on laboratory studies, which have demonstrated that a trait is cooperative. The gold-standard test for a cooperative gene involves a wild-type strain that produces the traits and a mutant strain that does not. If a trait is cooperative, then the producer will outperform the nonproducer when each is grown clonally, but nonproducers will outperform producers in groups (West et al., 2006). As an example, we look at the first gene on the list, *bslA* (formerly known as *yuaB*), which is involved in biofilm formation, and specifically in making the biofilm hydrophobic to resist chemical attack (Arnaouteli et al., 2017). A nonproducer of *bslA* cannot form normal biofilms on its own, but can get into mature biofilms when in co-culture with producers (Ostrowski et al., 2011). Further work mixing producers and nonproducers at a range of starting ratios demonstrated that the biofilm can maintain function as long as >50% of cells are producers (Arnaouteli et al., 2017).

The full list of cooperative genes can be found in Supplementary Table 2.

For the robustness check of whether deleterious mutations are over- or under-represented in cooperative genes, we used the protein localization tool PSORTb 3.0 (Yu et al., 2010). We categorize cooperative genes as those which PSORTb predicts to be extracellular. We also follow previous studies in removing genes for which PSORTb cannot make a definitive prediction (Dewar et al., 2021).

### Controlling for conditional expression

Conditional expression can lead to the same signatures of relaxed selection as kin selection for cooperation. We directly examined expression rates for the set of genes regulated by QS, using data from (Futo et al., 2021), who measured gene expression of >4,000 genes at 11 time points during biofilm formation.

For any pair of genes, we can calculate the correlation in gene expression across the 11 time points of the biofilm. For the 178 QS genes in our data set, there are 15,753 unique pairs of genes. The mean pairwise Spearman’s correlation in gene expression is .302. To test whether this set of genes is more or less correlated than a randomly chosen set of genes, we use a bootstrap approach. We take a random set of 178 genes and calculate mean pairwise correlation in the same way as before. Then we repeat 10,000 times. We find that the correlation in expression of our QS-controlled genes is higher than in 99.7% of our bootstrap samples (Supplementary Figure 3), demonstrating that our candidate set of genes is appropriate for our analysis of signature of selection.

### Other cooperative traits

We also examined five other types (groups) of traits, where we could compare genes for putatively private and cooperative traits, which are likely to be expressed at similar rates (Table 1; Figure 4). First, we used iron-scavenging via siderophores, which is a well-studied cooperative trait that is important for growth and survival of bacteria (Griffin et al., 2004; Kümmerli et al., 2015). Specifically, we looked at the *B. subtilis* siderophore bacillibactin (Miethke et al., 2006; Pi & Helmann, 2017). We classified the genes for biosynthesis bacillibactin as cooperative, and genes for the uptake and release of bound bacillibactin as private (Supplementary Table 3). We classified the genes for biosynthesis bacillibactin as cooperative and genes for uptake and release of bound bacillibactin as private (Supplementary Table 3).

**Table 1.** Traits used for comparisons of cooperative versus private genes The full gene lists are in Supplementary Tables 2–7.

Trait	Private genes	Cooperative genes
1. Quorum sensing traits	Genes that only affect the fitness of the producing cell (*N* = 25)	Genes for public goods that potentially provide benefits to the local group of cells (*N* = 153)
2. Iron scavenging	Genes for uptake and use of iron via the *B. subtilis* siderophore bacillibactin (*N* = 5)	Genes for biosynthesis and secretion of bacillibactin (*N* = 5)
3. Antimicrobial resistance	Genes for ABC transporters involved in multidrug resistance. These genes transport the intact antibiotic outside the cell (*N* = 11)	Genes for enzymes that deactivate beta-lactam and aminoglycoside antibiotics, providing cooperative benefits to other cells (*N* = 3)
4. Proteases	Genes for intracellular proteases, which are likely to be involved in processing and regulation of proteins within the cell (*N* = 9)	Genes for extracellular proteases, which are likely to be involved in collective feeding and motility (*N* = 8)
5. Toxins	Genes for contact-dependent LXG toxins, which are delivered by a Type VII secretion system (*N* = 6)	Genes for the diffusible secreted toxin bacilysin, which is activate against a broad range of bacterial competitors (*N* = 8)
6. Antimicrobial activity	Genes for the secondary metabolite bacillaene, which defends against predation by *M. xanthus* (*N* = 13)	Genes for the secondary metabolite plipistatin, which attacks fungal competitors (*N* = 4)

**Figure 2.**
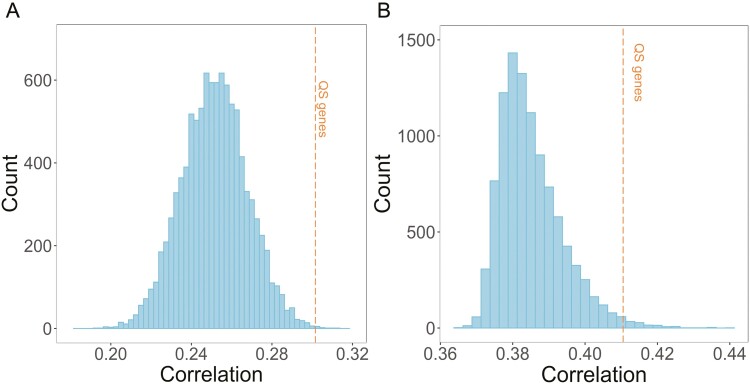
Average correlation in gene expression between genes during biofilm growth. (A) Correlation across 11 time points of biofilm formation for randomly sampled gene sets of the same size as our quorum sensing-controlled genes (*N* = 178). Data from Futo et al. (2021). (B) Correlation across eight time points of biofilm formation for randomly sampled gene sets of the same size as our quorum sensing-controlled genes (*N* = 160). Data from Pisithkul et al. (2019). In both panels, the orange line shows the average correlation for the quorum sensing-controlled genes, which is >99.7% of our random samples for (A) and >98.5% of random samples for (B).

**Figure 3.**
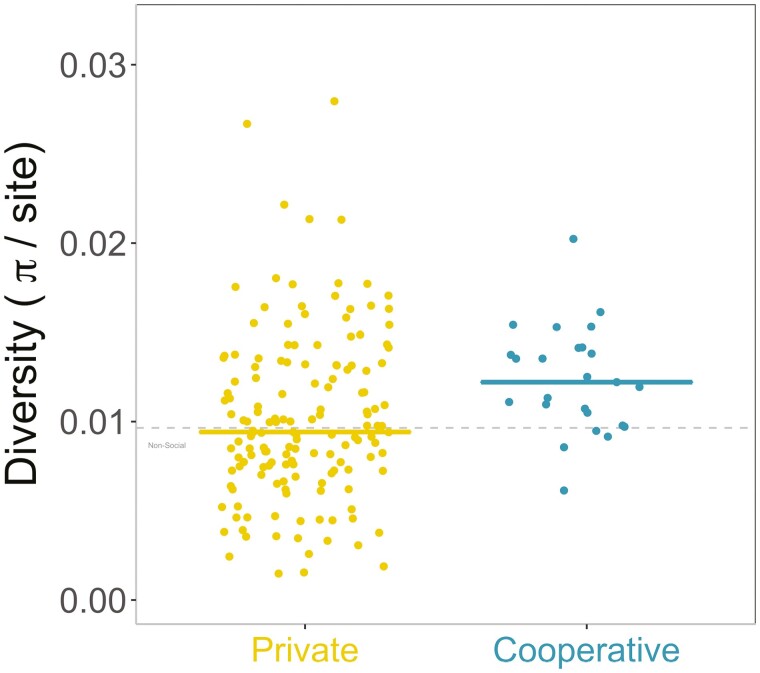
Nucleotide diversity per site for private (yellow) and cooperative (blue) genes controlled by quorum sensing. Each point is a gene, and the horizontal line shows the median for each group. The gray line shows the median for background private genes across the genome.

Second, we looked at antibiotic resistance genes. There are many mechanisms of antibiotic resistance, some of which are cooperative. For example, the secretion of beta-lactamases is a cooperative trait as they detoxify the external environment, providing benefits to the local population (Amanatidou et al., 2019; Dugatkin et al., 2005; Frost et al., 2018; Wang et al., 2023). We also classified aminoglycoside resistance as cooperative, as the modification of the antibiotic detoxifies the local environment (Poole, 2005). For the private genes, we used the eight ABC transporters that are thought to be involved in multidrug resistance by pumping antibiotics outside the cell (Quentin et al., 1999; Torres et al., 2009) (Supplementary Table 4).

Third, we looked at the range of peptidase proteases produced by *B. subtilis*. The functions of these proteases are broad, covering processing, regulation, and feeding, but we can separate them into cooperative and private genes by looking at those that are secreted (i.e., are extracellular) and those that are not secreted (Harwood and Kikuchi, 2022). The secreted proteases are more likely to have cooperative fitness effects on other cells, through nutrition, interacting with host immune systems etc. (Supplementary Table 6).

Fourth, we looked at toxins. For the cooperative genes, we used bacilycin, which is a secreted antimicrobial peptide that is active against a range of bacteria (Ertekin et al., 2020). Because bacilycin can diffuse through the environment, it likely has cooperative fitness effects on others. *Bacillus subtilis* also has many toxins that are involved in contact-dependent inhibition, and therefore likely have private effects on fitness. For the private gene, we used six LXG toxins (Kobayashi, 2021), which are delivered by a Type VII secretion system. While these toxins can still have cooperative effects by removing competitors, they are by their nature less cooperative than secreted molecule such as bacilycin. Both of these sets of genes are under the control of the DegS–DegU system (Supplementary Table 5).

Fifth, we looked at antimicrobials. *B. subtilis* produce a series of antimicrobial molecules, which vary in which organisms they target, how they act, and how they are secreted. We can however distinguish between antimicrobials that have a more defensive role in traits such as predation avoidance and those that have a more offensive role in competition with other species. While both of these categories likely have some component of cooperative and private effects on fitness, the offensive ones will be relatively more cooperative. This is because defensive molecules have a stronger effect on the individuals producing them (Supplementary Table 7).

### Identifying deleterious mutations

We used the variant annotation tool SnpEff (Cingolani et al., 2012) to look for SNPs that generate deleterious mutations in our data set. Specifically, we annotate two types of mutations: (a) premature stop codons and (b) frameshift mutations. This gives us a list of genes that have at least one deleterious mutation. To test whether a given set of genes are overrepresented for deleterious mutations, we use two percentages: (a) the % of genes in the whole genome that are in that set and (b) the % of genes with deleterious mutations that are in this set. We compare these values using a binomial test with the null hypothesis that the number of deleterious mutations in the gene set is equal to that expected by the frequency of the gene set. For any given gene set, we conduct a further test where we use the total number of deleterious mutations in that set of genes, rather than just the presence/absence of deleterious mutations for that gene.

### Statistics and figures

We conducted all statistical analysis in R (R, 2020). For the main statistical analysis comparing molecular population genetic parameters of cooperative and private genes, we use one of two statistical tests, depending on the variable in question. Some of the molecular population genetic parameters we calculate are normally distributed, but others tend to be highly skewed. The skewed variables cannot be transformed into normally distributed, so we use different statistical tests. For variables that are normally distributed, we used an ANOVA to compare the three groups of genes. Because of unequal sample sizes, we used Welch ANOVA that does not require equal variance. We used the Games–Howell post hoc test, which is similar to Tukey’s HSD, but designed for Welch ANOVA where we do not have to assume equal variance. For variables that are not normally distributed, we used the Kruskal–Wallis test, which compares medians. We then used the Dunn test for post hoc comparisons of groups.

**Figure 4.**
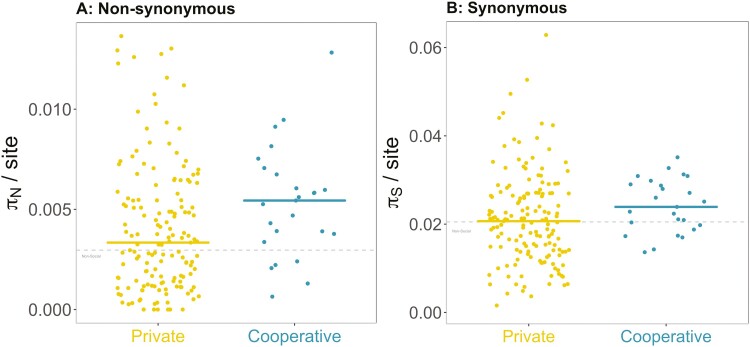
Nucleotide diversity at nonsynonymous (A) and synonymous (B) sites for private (yellow) and cooperative (blue) genes controlled by quorum sensing. Each point is a gene, and the horizontal line shows the median for each group. The gray line shows the median for background private genes across the genome.

All results figures were made using the ggplot2 package in R (Wickham, 2016) using color palettes from the packages wesanderson (github.com/karthik/wesanderson) and BirdBrewer (https://github.com/lauriebelch/BirdBrewer). Figure 4 illustrating the secondary comparisons was made using BioRender.

### Bioinformatics

Raw reads for each of the 31 strains were downloaded from the European Bioinformatics Institute’s European Nucleotide Archive (accession number PRJEB43128). We then used an SNP calling pipeline to find SNPs in each strain compared to the reference NCIB 3610 (accession NZ_CP020102.1).

### Trimming and quality control

We used Trimmomatic to remove adapters remove low-quality reads, which we did by removing leading and trailing reads if the quality score was <3 or if average quality in a four-base sliding window was <20. We manually checked the output of this step using the reports produced by FastQC (Andrews, 2010).

### Mapping

We used BWA (Li & Durbin, 2009) to map reads from each strain to the reference strain. We used SAMtools (Li et al., 2009) to convert the mapping files from BAM to SAM and used Picard tools (Broad Institute, 2019) to remove PCR duplicates.

### Variant calling

We used BCFtools (Danecek et al., 2021) to call variants on all strains and produce a VCF file that can be read by R for population genetic analysis.

### Filtering and quality control

We conducted further filtering to remove indels, and filter for mapping quality, read depth, and strain bias using the default setting of SAMtools vcfutils python script. We then removed all sites which hadn’t been called in at least 80% of strains. We also used the coverageBed tool in BEDtools (Quinlan & Hall, 2010) to record the percentage of each gene length that had been mapped, in order to adjust per-site measures to the correct length. After filtering, we had a total of 256,769 SNPs among the 31 strains.

### Outgroup

We used the phylogeny in Kalamara (2021) (the source of these strains) to identify *Bacillus subtilis subsp. spizizenii* str. W23 as an appropriate outgroup (accession NC_014479.1, raw sequencing data SRR2063059). We used the same variant calling pipeline as above to produce a second VCF which included the SNPs from the outgroup.

### Population genetic measures

We used the PopGenome package from R (Pfeifer et al., 2014) to conduct the main molecular population genetic analysis. All parameters were scaled to the corresponding mapped gene length, and any gene with mapped length <50% of their full length or lacking polymorphism data was removed from the analysis, leaving 3,817 genes for the population genetics analysis. Using PopGenome, we calculated Nucleotide polymorphism, Tajima’s *D*, Fu and Li’s D*, the Mcdonald–Kreitman *p*-value, Direction of Selection statistic, and neutrality index. We also calculated separate measures for synonymous and non-synonymous sites where appropriate.

To calculate divergence, we measured the rate of protein evolution *K*_a_/*K*_s_ by comparing the reference strain to the outgroup. We did this by creating a pseudogenome of the outgroup by inserting the relevant SNPs into the reference sequence using the GATK suite of tools (McKenna et al., 2010). This pseudogenome could then be read by R, and we used the seqinR package (Charif & Lobry, 2007) to calculate divergence.

## Results and discussion

We compared genes controlling traits that are hypothesized to be cooperative, with traits that are hypothesized to be private. We identified six different types of putatively cooperative behavior, where an appropriate comparison could be made with genes controlling private traits, which are likely to be expressed at similar rates (Table 1). We used the first type, QS, for the main analysis, and we summarized the other five types at the end of the Results and discussion section.

### Quorum sensing

We started by examining genes induced by the ComQXPA QS signaling system (Arnaouteli et al., 2021; Kalamara et al., 2018). This system regulates gene expression in response to the density of a diffusible signal molecule. At high cell densities, the density of the signal molecule increases, causing ComA to activate, and the upregulation of a number of traits (Comella & Grossman, 2005; López & Kolter, 2010; Nakano et al., 1991; Špacapan et al., 2020). We categorized genes as cooperative or private based on a search of the literature on *B. subtilis* (Methods). For example, the 15 genes coding for the exopolysaccharide EPS are classed as cooperative. EPS is the main biofilm matrix component (Branda et al., 2001) and is required for biofilm formation (Branda et al., 2004). EPS is costly to produce, it provides benefits to nonproducers, and nonproducers can exploit producers (Van Gestel et al., 2014). This is a classic public good. Similarly, TasA, a protein fiber that is needed for biofilm structural integrity (Erskine et al., 2018; Romero et al., 2010), has also been shown in lab experiments to be a public good (Dragoš et al., 2018). Mutants lacking genes for either EPS or TasA can also complement each other, providing further evidence that the benefits of these genes are shared (Branda et al., 2006). Private genes include those coding for traits such as asparagine synthase (AsnB), which controls peptidoglycan hydrolysis for cell growth and cell-wall synthesis (Yoshida et al., 1999). We found that QS controls a mixture of private and cooperative traits in *B. subtilis*, categorizing *N* = 25 of our QS-controlled genes as cooperative, and *N* = 153 as private (Supplementary Table 2).

We started by focusing on QS because it offers a number of advantages for our purpose. First, the large size and nature of this network means that there are sufficient private and cooperative genes for a targeted analysis (*N* = 153 and *N* = 25, respectively). Second, shared control by the same signaling system means that private and cooperative genes controlled by the QS system are likely to be co-expressed at the same time (Azimi et al., 2020a; Rutherford & Bassler, 2012). Third, there are data on gene expression allowing us to directly look at co-expression rates (Futo et al., 2021). Fourth, the coregulation of genes acts as a control for mutations in noncoding regulatory and promoter regions that could affect the production of cooperative public goods.

### QS: testing if genes are co-expressed at the same time

Differential gene expression can also influence the strength of selection and so needs to be accounted for. Theory tells us that the fraction of generations in which a trait is expressed can determine the extent to which selection is relaxed (Van Dyken & Wade, 2010). We therefore need to compare genes that are switched on or off in the same conditions. Shared control by the same signaling system means that private and cooperative genes controlled by the QS system are highly likely to be co-expressed (Azimi et al., 2020a; Rutherford & Bassler, 2012). Furthermore, many genes regulated by QS form operons that share a promoter and are transcribed and translated together (Kalamara et al., 2018). We were, however, also able to test this assumption directly by examining two data sets on gene expression (Futo et al., 2021; Pisithkul et al., 2019).

Futo et al. (2021) measured gene expression at 11 different points in the formation of a biofilm, following normal practice by normalizing their results to the median to convert expression levels to the same scale. This gives us an excellent data set, as we can use simple correlations between pairs of genes to see whether they are up- and downregulated at the same time. For our 178 private and cooperative genes, there are 15,753 unique pairs of genes. The average correlation in gene expression for a pair of these genes is .302 (Spearman’s correlation). We then used a bootstrap approach to see if this correlation was greater or lower than for randomly chosen genes. We took a random set of 178 genes and calculate mean pairwise correlation in the same way and repeated this process 10,000 times. We found that the mean pairwise Spearman’s correlation was .251 for the random (bootstrap) samples and that the correlation in expression of our QS-controlled genes was higher than 99.7% of our bootstrap samples (Figure 2). Consequently, our candidate set of genes has expression rates that are correlated significantly higher than expected by chance, supporting our choice for their use in an analysis of signature of selection (*p* < .004). This is unsurprising, given that many of our QS genes are clustered together as operons, while the randomly sampled genes are unlikely to be. However, for our purposes we only need to test whether genes are co-expressed, we do not need to control for factors like operons that might explain *why* they are co-expressed.

Pisithkul et al. (2019) provided a different data set measuring gene expression in biofilms. Whereas Futo et al. measured expression over 2 months in a biofilm with a solid–air interface, Pisithkul et al. focused on the initial stages of biofilm growth, measuring expression over 24 hr in a biofilm with a liquid–air interface. Using this second data set, we again found that QS-controlled genes have expression rates that are correlated significantly higher than expected by chance (*N* = 160 genes, *p* < .02; Supplementary S11).

### QS: polymorphism

Polymorphism (π) is measured as the average number of pairwise nucleotide differences per site in a gene. We found that genes for putatively cooperative traits had significantly greater polymorphism than genes for private traits (Figure 3; ANOVA *F*_2,61_ = 11.82, *p* < .001; Games–Howell test *p* < .001; *N* = 25 cooperative genes, *N* = 153 private genes). Cooperative genes also had significantly greater polymorphism when we only examined nonsynonymous sites (Kruskal–Wallis χ^2^(2) = 10.7, *p* < .01; Dunn test *p* = .0240. Figure 4A) or only synonymous sites (ANOVA *F*_2,61_ = 7.30, *p* <.001; Games–Howell test *p* = .007) (Figure 4B). The increase in polymorphism that we see in cooperative traits does not imply an increase in cheating, as it is just the consequence of the relaxation of selection.

The trend for greater synonymous polymorphism is possibly surprising as such sites should be under much weaker selection, and we would not necessarily expect to see an effect of kin selection. However, we also found this pattern in *P. aeruginosa* (Belcher et al., 2022). There is some evidence that differences in the use of preferred codons between cooperative and private genes could explain this pattern (Supplementary S1). Synonymous mutations can also have substantial fitness effects on social traits (Azimi et al., 2020b; Meir et al., 2020; Salverda et al., 2010; Zwart et al., 2018). Because we focus on the relative level of polymorphism, features of *B. subtilis* such as spore-formation cannot explain these patterns unless they only affect cooperative genes.

The ratio between nonsynonymous and synonymous polymorphism does not differ between cooperative and private genes (Kruskal–Wallis χ^2^(2) = 6.97, *p* = .0306; Dunn test *p* = .183. Supplementary Figure 1), which reflects the fact that cooperative genes have elevated diversity at both types of site, possibly due to the large fitness effects of many traits (both cooperative and private) that are QS controlled (Schuster et al., 2017; Whiteley et al., 2017) (Supplementary S2).

As an additional control, we were able to repeat all of these analyses comparing cooperative QS genes against a different group of private genes not controlled by QS (*N* = 1,832). This different set of *N* = 1,832 private genes, which we call “background genes” are those that are not controlled by QS and whose products are found in the cytoplasm, where they are least likely to have a cooperative function. In all cases, we found the same pattern, with cooperative QS genes showing elevated polymorphism compared to background genes (Supplementary S2).

### QS: divergence

**Figure 5.**
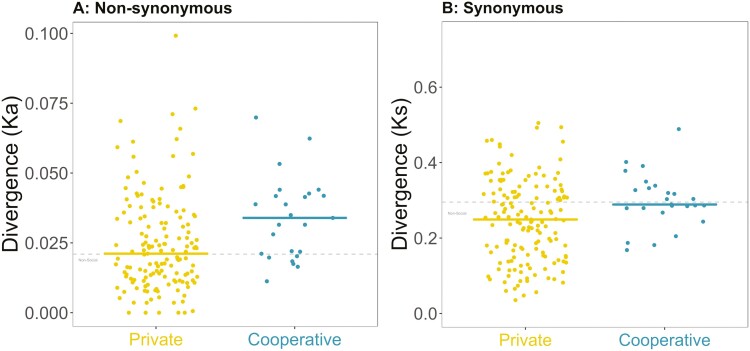
Divergence at nonsynonymous (A) and synonymous (B) sites for private (yellow) and cooperative (blue) genes controlled by quorum sensing. Divergence is measured by rates of protein evolution, for example, number of synonymous substitutions per synonymous site for (B). Each point is a gene, and the horizontal line shows the median for each group. The gray line shows the median for background private genes across the genome.

We found that cooperative genes had significantly greater divergence compared to private genes at both nonsynonymous and synonymous sites (nonsynonymous: Kruskal–Wallis χ^2^(2) = 10.4, *p* = .006; Dunn test *p* = .00553; Figure 5A; synonymous: ANOVA *F*_2,59_ = 7.26, *p* < .01; Games–Howell test *p* = .011; Figure 5B). We examined synonymous and nonsynonymous sites separately because we measure divergence through rates of protein evolution. A signature of selection can be found at both synonymous and nonsynonymous sites, implying that synonymous variation is not neutral (Belcher et al., 2022; de Oliveira et al., 2019). Because divergence is elevated at both types of sites (similar to polymorphism), there is no difference in the ratio of nonsynonymous to synonymous divergence (Kruskal–Wallis χ^2^(2) = 13.32, *p* = .00128; Dunn test *p* = .189. Supplementary Figure 2), although both sets of QS genes have a higher ratio than the background genes (Supplementary S2). This could reflect stronger selection of both private and cooperative QS traits compared to background traits.

### Alternative hypotheses

We were also able to eliminate alternative explanations for the patterns that we observed with both polymorphism and divergence (Figures 3–5). Greater polymorphism would also have been expected if cooperative genes were more likely to be experiencing balancing or frequency-dependent selection, while greater divergence would arise from positive/directional selection leading to fixation of adaptive mutations (Linksvayer & Wade, 2009, 2016). Alternatively, the pattern we observed could have been caused by other differences between putatively cooperative and private genes. We assessed these alternative hypotheses by testing other predictions that they make.

First, we found no evidence that cooperative genes were more likely to be under balancing selection, which would lead to a deficit of rare alleles in the population (Tajima’s *D*, Fu and Li’s *F*, Fu and Li’s *D*, Supplementary S3). Second, we found no evidence that cooperative genes are more likely to be under positive selection, which would lead to an excess of nonsynonymous divergence compared to nonsynonymous polymorphism, as adaptive mutations would quickly spread and only be detected as divergence. We test for this with the commonly used Mcdonald–Kreitman test and downstream analysis (Neutrality Index and Direction of Selection statistic), and also a modified version of the McDonald–Krietman test that is more conservative and deals better with non-neutrality of synonymous mutations (Supplementary S4). Third, we found no evidence that the patterns observed are due to a lack of statistical power, or any differences in gene length or likelihood of horizontal gene transfer between cooperative and private genes, or a lack of statistical power (Supplementary S5). Fourth, we found no evidence that our results could be explained by noise due to variation in the recombination rate. The set of strains that we analyzed vary in genetic competence, the ability to take up DNA from the environment (Kalamara et al., 2021), which is a form of recombination in bacteria. This variation in competency could create noise in our population genetic measures that focus on SNPs, due to the variation in recombination. Considering the 31 strains we analyzed, 18 are genetically competent (Kalamara et al., 2021). We conducted an analysis of polymorphism and divergence using only the competent strains and found the same patterns as when we use all strains (Supplementary S7). Fifth, we found the same signature of selection when analyzing operons as independent data points, rather than genes (Supplementary S12). Sixth, we found the same signature of selection when removing the small number of essential genes that are regulated by QS in this species (Commichau et al., 2013; Supplementary S13). Seventh, we found that cooperative and private genes do not differ in their maximum expression level (Supplementary S14), which is another factor known to predict selection on genes (Urrutia & Hurst, 2001, 2003).

Finally, we found no evidence that the patterns we observed could be caused by division of labor (Cooper & West, 2018; Cooper et al., 2021; Liu et al., 2021; West & Cooper, 2016), which is a cooperative hypothesis not mutually exclusive with kin selection. In *B. subtilis* biofilms, some cells will produce the polysaccharide EPS, and some will produce TasA amyloid fibers (Chai et al., 2008). Mutants lacking one or the other cannot grow alone but can grow together (Dragoš et al., 2018). The extra level of conditional expression in these public goods (over that caused by being QS controlled) could leave a signature of relaxed selection that is not caused by kin selection. To investigate this possibility, we took advantage of the fact that this heterogeneity is ultimately caused by Spo0A. Spo0A is a bistable switch that is active in only a subset of cells (Chai et al., 2008; González-Pastor, 2011) and activates SinR antirepressors which control the operons for both EPS and TasA (Dragoš et al., 2018; Kobayashi, 2008; Molle et al., 2003). If the extra conditionality of division of labor was causing an effect, we would expect that genes under the control of Spo0A (N = 20) would have greater polymorphism than other QS-controlled genes (*N* = 157), but this is not the case (Supplementary S6). We note that the cooperative genes EPS and TasA that are known to have a division of labor both stand out as highly polymorphic within the genes controlled by Spo0A, providing support to our hypothesis that sociality causes the effect. Overall, we can rule out any confounding effect of the extra level of conditionality in some cooperative traits (Supplementary S15).

### Other cooperative traits

**Figure 6.**
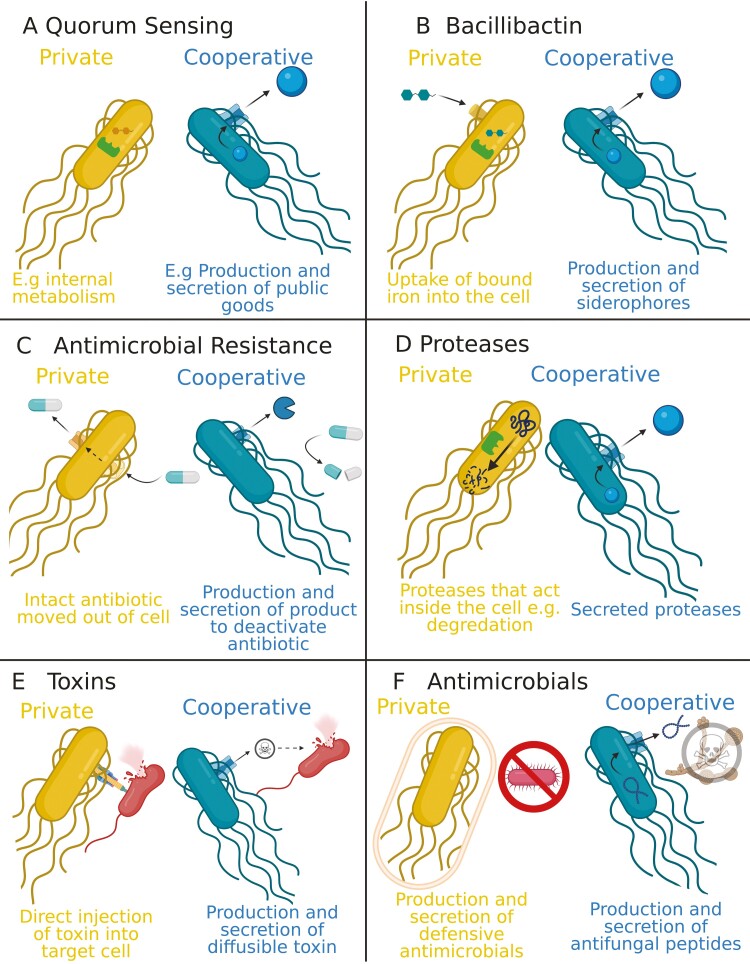
Diagram of how traits are categorized as either private (yellow) or cooperative (blue). (A) Categorization of quorum sensing-controlled traits for the main analysis, with private traits giving fitness benefits only to those expressing the gene, and cooperative traits giving fitness benefits that can potentially be shared with other cells. (B–F) Other cooperative traits: (B) bacillibactin (iron scavenging), (C) antimicrobial resistance, (D) proteases, (E) toxins, (F) antimicrobials. Note that for some of these comparisons it is the relative level of sociality that is different. Figure created with BioRender.com.

Our above analyses have provided strong evidence for relaxed selection due to kin selection for cooperation, considering genes controlled by QS. We then tested if the same pattern was found in five other types (groups) of traits, where we could compare genes for putatively private and cooperative traits (Table 1; Figure 6). These genes are likely to be expressed at similar rates, which controls for the confounding effect of conditional expression. The extent to which the distinction between private and cooperative traits can be made varies across these other groups of traits. Consequently, we might not expect to see a signature of kin selection in every case, and so our aim is to see if there is a relatively consistent pattern.

First, *B. subtilis* produces and secretes a siderophore named bacillibactin, which binds to iron in the environment (Miethke et al., 2006; Pi & Helmann, 2017) (Figure 6B). The bound complex can be taken into the cell, but is also available to nonproducers, and is therefore a public good. We separated the genes involved in the bacillibactin pathway into cooperative and private components, with genes involved in biosynthesis and export classed as cooperative, and those involved in uptake and release of bound iron classed as private. This is our strongest comparison, as epistatic interactions mean that production and uptake are necessarily linked. Second, *B. subtilis* exhibits resistance to antimicrobials by either pumping intact antibiotics outside the cell (private) or producing enzymes such as beta-lactamases that detoxify the environment for the entire community (Bucher et al., 2019; Noguchi et al., 1993; Torres et al., 2009) (cooperative; Figure 6C). Third, *B. subtilis* produces proteases to break down proteins, with different proteases acting either inside the cell (private) or secreted to act outside the cell (Harwood & Kikuchi, 2022; Koo et al., 2017; Pohl et al., 2013) (cooperative; Figure 6D). Fourth, *B. subtilis* produces toxins that can either be contact dependent (relatively private) or diffusible throughout the community (cooperative public goods; Figure 4D). However, this comparison is relative, and possibly weak, as killing cells with contact-dependent toxins could also provide a cooperative benefit to other local cells, which experience reduced competition (McNally et al., 2017). Fifth, *B. subtilis* has a number of antimicrobial traits that are more defensive against predators without affecting the predators’ growth (private) and those that are more offensive against competitors (cooperative) (Müller et al., 2014; Romero et al., 2007). This is also a weak comparison, as bacillaene (the defensive molecule) is secreted from cells, and so likely also has some cooperative component. However, the defensive traits provide a relatively more private benefit in providing personal protection, whereas the removal of direct competitors by the offensive traits provides a relatively more cooperative benefit. For all comparisons, we find that the set of genes has significantly correlated expression, using the same methodology and data as for the comparison with QS genes (Supplementary S9) (Futo et al., 2021).

The number of cooperative genes was too small to analyze each case separately, for example, the iron-scavenging comparison involves only 10 genes (5 private and 5 cooperative). Consequently, we examined the data in three ways. First, we grouped all cooperative genes together into one set (*N* = 52) and compared them to the grouped private genes across all comparisons (*N* = 194). Second, we grouped all of the cooperative genes from just the five new comparisons (i.e., not QS) (*N* = 27), and compared them to the private genes from these five comparisons (*N* = 41). Third, we consider each of the six categories of cooperative versus private genes (Table 1) as its own data point (*N* = 6).

### Other cooperative traits: polymorphism and divergence

**Figure 7.**
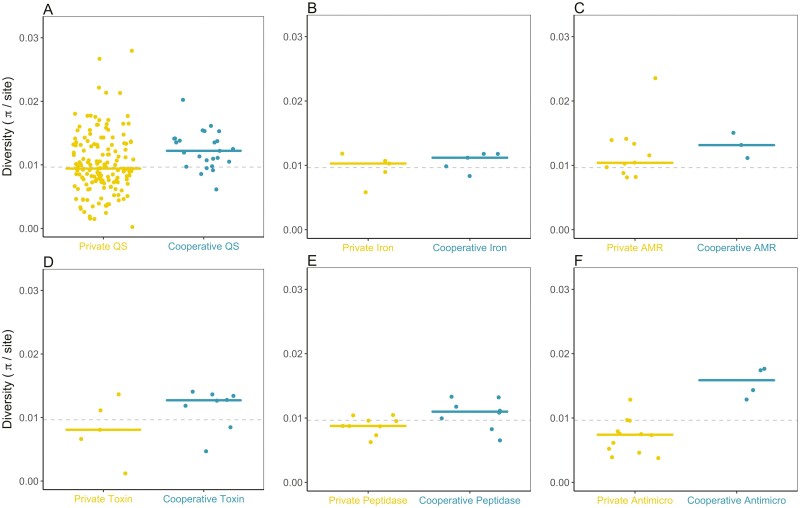
Private (yellow) versus cooperative (blue) polymorphism in genes for six traits. (A) The quorum sensing-controlled genes used in the main analysis. (B–F) The other cooperative traits.

We found that polymorphism was consistently higher in cooperative genes than in private genes (Figure 7). This pattern is consistent across the three different ways that we can analyze our data: All gene comparison (ANOVA *F*_2,127_ = 10.59, *p* < .0001; Games–Howell test *p* ≤ .001); just the genes for the social traits other than QS (ANOVA *F*_2,47_ = 5.94, *p* < .001; Games–Howell test *p* < .01); and the six-category comparison (Wilcoxon signed-rank test *V* = 21, *p* = 0031).

Polymorphism at nonsynonymous sites is also significantly higher in cooperative genes than in all private genes. This pattern is consistent across the three different ways that we can analyze our data: All genes comparison (Kruskal–Wallis χ^2^(2) = 19.71, *p* < 10^−4^, Dunn test *p* < 10^−4^); just the genes for the social traits other than QS (Kruskal–Wallis χ^2^(2) = 9.90, *p* < .01, Dunn test *p* < .01); and the six-category comparison (Wilcoxon *V* = 21, *p* = .031) (Supplementary Figure 3).

Polymorphism at synonymous sites is also significantly higher in all cooperative genes than in all private genes when analyzing all genes (ANOVA *F*_2,130_ = 4.21, *p* = .016; Games–Howell test *p* = .019). However, this trend was not significant when examining just the genes for the social traits other than QS (ANOVA *F*_2,47_ = 2.28, *p* = .113; Games–Howell test *p* = .10) or when using categories as data points (synonymous polymorphism is marginally higher in private genes than cooperative genes for the iron-scavenging and AMR categories; Wilcoxon *V* = 18, *p* = .156). We would expect the pattern to be weaker with synonymous polymorphism, as these sites are likely to be under weaker selection.

Nonsynonymous divergence is significantly greater in all cooperative genes compared to all private genes. This pattern is consistent across the three different ways that we can analyze our data: All genes comparison (Kruskal–Wallis χ^2^(2) = 22.9, *p* < 10^−4^, Dunn test *p* < 10^−4^); the genes for the social traits other than QS (Wilcoxon *V* = 21, *p* = .031 Supplementary Figure 4); and the six-category comparison (Kruskal–Wallis χ^2^(2) = 14.7, *p* < .001, Dunn test *p* < .001). As would be expected, the pattern is more mixed for synonymous divergence. While synonymous divergence is significantly greater in all cooperative genes compared to all private genes (ANOVA *F*_2,125_ = 8.33, *p* = .001; Games–Howell test *p* < 001), this is not the case when examining just the genes for the social traits other than QS (ANOVA *F*_2,45_ = 2.10, *p* = .013; Games–Howell test *p* = .39) or the six-category comparison (Wilcoxon *V* = 13, *p* = .688) (Supplementary Figure 6). Similarly, the ratio between nonsynonymous and synonymous divergence is significantly greater in all cooperative genes compared to all private genes (Kruskal–Wallis χ^2^(2) = 25.0, *p* < 10^−5^, Dunn test *p* = .012), but not when we just looked at the genes for the social traits other than QS (Kruskal–Wallis χ^2^(2) = 13.2, *p* < .01, Dunn test *p* = .011) or the six-category comparison (Wilcoxon *V* = 18, *p* = .156) (Supplementary Figure 7). This may reflect differences in selection on synonymous variation on different traits or the weakness of some of these comparisons due to small sample sizes. While the fact that *K*_a_ tends to increase in cooperative genes, but *K*_s_ stays close to background levels may be a sign of weak positive selection, we confirmed that none of our cooperative versus private comparisons show significant differences in balancing or positive selection (Supplementary S10), suggesting that what we are seeing is a signature of kin selection and that we may just lack power in our other comparisons.

Overall, these results incorporating other traits in addition to QS-controlled genes provide support to the main result that there is a signature of kin selection for cooperation (Figures 3–5). Nonetheless, the a priori distinction between private and cooperative traits is weaker for some comparisons, and we could expect exceptions within the overall pattern. The main exception in our analyses was toxin comparison, where we compared contact-dependent LXG toxins (*N* = 6 genes) to the secreted antimicrobial bacilycin (*N* = 8 genes). Both of these sets of genes are involved in competition in biofilms, and both are controlled by the DegS–DegU system, so likely expressed at the same time (Kobayashi, 2021; Mariappan et al., 2012). Possible confounding factors in this case include the fact that although we classified the contact-dependent toxins as private, they also provide cooperative benefits to local cells by eliminating competitors. The LXG toxins also stand out because three of them are on phage elements (Kobayashi, 2021), and it may be that the strength or type of selection is different on these genes, masking any effect of sociality.

### All traits: deleterious mutations

As an additional robustness test for our conclusions, we analyzed deleterious mutations, specifically those that cause loss of function, generate stop codons, or cause a frameshift. If kin selection is favoring cooperation, we should observe more deleterious mutations in genes controlling cooperative traits compared to private traits. This is because relaxed selection slows the rate at which deleterious mutations are purged from the population (Linksvayer & Wade, 2009, 2016; van Dyken & Wade, 2012). We tested this prediction by looking for deleterious mutations in our SNP data and repeated this analysis with two different data sets.

First, we used all cooperative genes from our six comparisons in Table 1. We measured how many cooperative and private genes have deleterious mutations and compared this to an expectation based on their relative frequency across the genome. Cooperative genes were significantly more likely than private genes to have deleterious mutations, χ^2^(1) = 12.3, *p* < .001. This pattern also holds if we count total number of deleterious mutations, rather than just presence or absence, χ^2^(1) = 11.0, *p* < .001.

To test the robustness of this result, we repeated the analysis using the localization prediction tool PSORTb to categorize genes for extracellular proteins as “cooperative” and genes for proteins that are not secreted as “private” (Yu et al., 2010). This method has been previously used in many studies to estimate whether genes are for cooperative (social) or private traits (Dewar et al., 2021; Garcia-Garcera & Rocha, 2020; Nogueira et al., 2009, 2012). By using PSORTb, we are able to systematically analyze all genes, which increases our sample size and statistical power compared to the “artisan” approach we use in the main analysis. We removed 17% of all genes with unknown localization, leaving us with a set of genes of known sociality. We found deleterious mutations in 293 genes, of which 17 are cooperative (5.8%). This is significantly more than expected given that cooperative genes only make up 2.0% of genes (binomial test *p* < .001), matching our prediction that deleterious mutations should be biased toward cooperative genes. If we count total deleterious mutations (rather than number of genes with at least one), we see the same pattern, with cooperative genes making up 5.5% of mutations (20 of 361).

### Relatedness estimation

The genetic relatedness between interacting cells (*r*) is a key parameter for social evolution. Relatedness can be very hard to estimate for natural populations of bacteria and other microbes, except in extreme cases where interactions take place in some physical structure such as a fruiting body or a filament (Flowers et al., 2010; Gilbert et al., 2012). Population genetic data allow relatedness to be estimated indirectly because the degree to which selection is relaxed, and greater polymorphism will be observed, depends on the relatedness between interacting cells. Consequently, if we assume that the patterns of polymorphism and divergence that we see are due to relatedness being <1, then we can work backwards from the polymorphism data to obtain an indirect estimate of relatedness (Belcher et al., 2022). This approach also requires assumptions about selection coefficients. As in previous studies, we have to assume that the magnitude of selection and the distribution of selection coefficients is the same on average for cooperative and private genes, which we cannot be sure is true. We also assume that we have identified the relevant private genes to compare our cooperative genes to. In *P. aeruginosa*, environmental isolates differ considerably in which genes are controlled by QS (Chugani et al., 2012), and the identity and number of genes can readily evolve under experimental evolution (Smalley et al., 2022). We also have to calculate relatedness across the whole genome because our calculation involves comparing the median polymorphism in cooperative genes to the median polymorphism in private genes. Although the correct measure of relatedness is that of the locus which controls the trait (West et al., 2006), this should correlate with whole-genome similarity. With these caveats in mind, by examining the polymorphism data from all genes, we estimated relatedness to be *r* = .79 (Supplementary S8).

An advantage of this indirect population genetic method for estimating relatedness is that it does not require knowledge about factors that would be hard or impossible to measure in natural populations. For example, the spatial details of how cooperative interactions play out, such as how far do public goods diffuse, and who benefits, as well as how much these vary in different environments, and the frequency with which different environments are encountered (Belcher et al., 2022; Nee et al., 2002). Indeed, cooperative traits in *B. subtilis* vary in the degree to which they are shared depending on whether groups are exhibiting sliding motility or growing in biofilms (Jautzus et al., 2022). In contrast, the indirect measure provided by population genetics represents an average of the different cooperative traits, over the different environments encountered, over evolutionary time. This population genetic approach represents an alternative or opposite way of looking at the data and so we should be careful not to overinterpret both at the same time—we can either test for relaxed selection to test for kin selection or we can look at the extent of relaxed selection to estimate relatedness.

## Conclusions

We have found strong evidence of kin selection for cooperation in a natural population of *B. subtilis.* Our analyses controlled for possible confounding factors, such as expression rate, and eliminated alternative explanations for polymorphism and divergence, providing evidence that complements the numerous lab experiments demonstrating sociality in this species (Branda et al., 2001, 2004; Chai et al., 2008; Dragoš et al., 2018; Erskine et al., 2018; Kraigher et al., 2021; Lyons et al., 2016; Martin et al., 2020; Romero et al., 2010; Stefanic et al., 2015; Van Gestel et al., 2014). Taken together with a previous study, population genetic analyses have now provided evidence of kin selection for cooperation in both gram-positive (*B. subtilis*) and gram-negative (*P. aeruginosa*) bacteria (Belcher et al., 2022). These results suggest convergent, and potentially widespread, kin selection for cooperation, based on very different underlying mechanisms, across bacteria.

A possible complication with studying cooperation in bacteria is that the extent to which traits are cooperative, and their importance for fitness, can depend greatly on environmental conditions (Connelly et al., 2017; Kümmerli et al., 2009; Sexton & Schuster, 2017; West et al., 2012; Xavier et al., 2011). One advantage of the molecular population genetic approach that we have used is that it averages across different environments over evolutionary time. Consequently, rather than examining a specific environment, it provides an “average” answer. In the case of *B. subtilis*, for the traits that we have examined, we have found evidence of kin selection for cooperation, with an estimated average relatedness of *r* = .79.

## Supplementary Material

qrad029_suppl_Supplementary_Material

## Data Availability

Data and code are available online at https://github.com/lauriebelch/bacillus.
