# Supplementary material for: Signatures of kin selection in a natural population of the bacteria Bacillus subtilis
Source: Evol Lett. 2023 Jul 18;7(5):315–30. doi: 10.1093/evlett/qrad029 (PMC10565896; doi:10.1093/evlett/qrad029)
Supplement: qrad029_suppl_Supplementary_Material [file qrad029_suppl_supplementary_material.pdf]

Supplementary Material

S1: Codon usage

In the main text, we showed that the elevated polymorphism in cooperative genes relative to private genes occurs at both synonymous and non-synonymous sites. This may be due to selection on synonymous codon usage, which is common across bacteria (1). In general, some codons may be preferred due to GC or AT preference, tRNA availability, or metabolic costs (2–5). We used the R package ‘sscu’ (6) to investigate whether codon usage differences between cooperative and private genes can explain the elevated synonymous polymorphism observed in social genes.

For some measures, we compare social genes to a set of highly expressed genes, under the assumption that highly expressed genes are under the strongest selection to optimize codon usage. We followed Rocha 2004 (5) in using ribosomal proteins as this set. For social genes, we used genes coding for extracellular proteins (determined by PSORTb), as this can be systematically applied to the whole genome.

The first measure we calculated is relative synonymous codon usage (RSCU). This gives a measure of the relative use of each codon, compared to the null expectation that each codon for an amino acid is used equally (RSCU =1) (Supplementary Figure S1.1). If cooperative genes had higher synonymous polymorphism due to codon usage, we would expect them to use each codon randomly (blue dots close to RSCU=1), whereas private genes might have a proffered codon for each amino acid (lots of yellow dots with high RSCU). This isn’t the case.

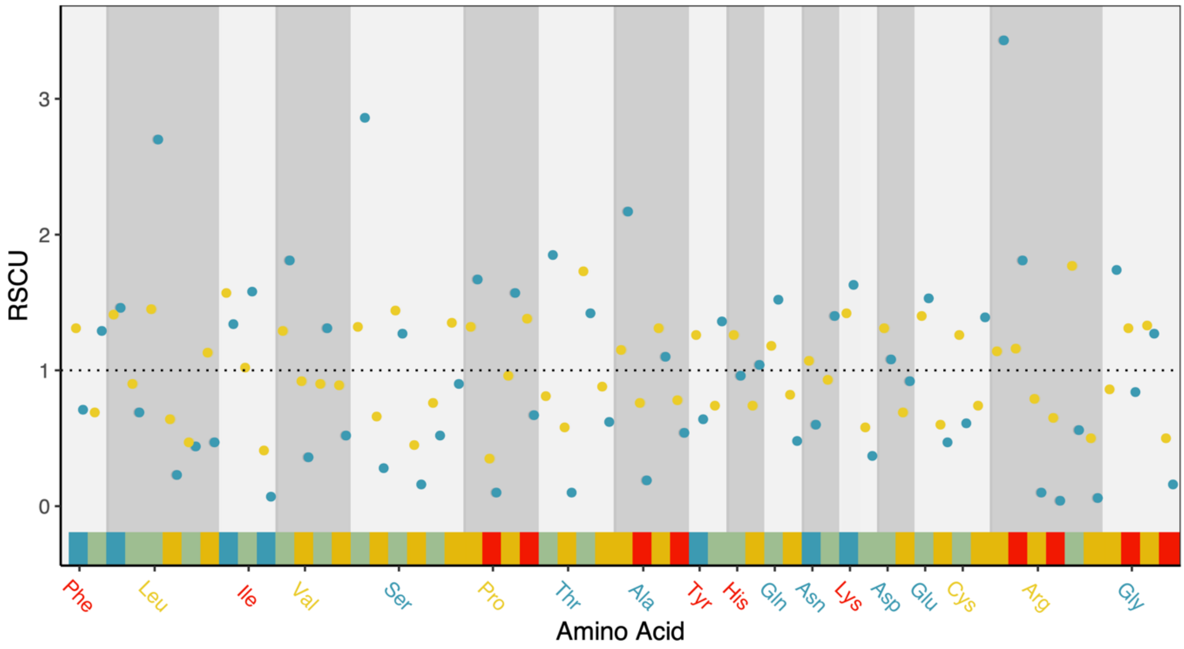

Supplementary Figure S1.1: RSCU for cooperative genes (blue) alongside highly expressed (ribosomal) genes (yellow). The dotted line shows RSCU=1, which is when each codon for an amino acid is equally likely to be used. If codons are used at random, we would expect all RSCU values to be close to 1. If certain codons are strongly preferred, then each amino acid should have a codon for which RSCU>>1. The colour bar shows the GC content of each codon, with red=3, yellow=2, green=1, blue=0. The colour in the label of each amino acid represents the biosynthesis cost in *E. coli*, with blue the lowest cost, yellow intermediate cost, and red high cost.

To determine if codon usage is more even in social genes or highly expressed genes, we calculate Shannon entropy for each amino acid. If each codon is used evenly, then Shannon entropy will be 0. If one codon is used more frequently than random, then Shannon entropy will be negative. This is analogous to how entropy is used in ecology to calculate species richness. An amino acid mostly being coded for with the same codon is equivalent to one species dominating in a species richness measure.

On average, Shannon entropy is much less negative for cooperative genes (-0.215 compared to -0.869 for highly expressed genes), suggesting that cooperative genes have much more even usage of codons, suggesting that there may be relaxed selection for synonymous codon usage in cooperative genes (Supplementary Figure S1.2)

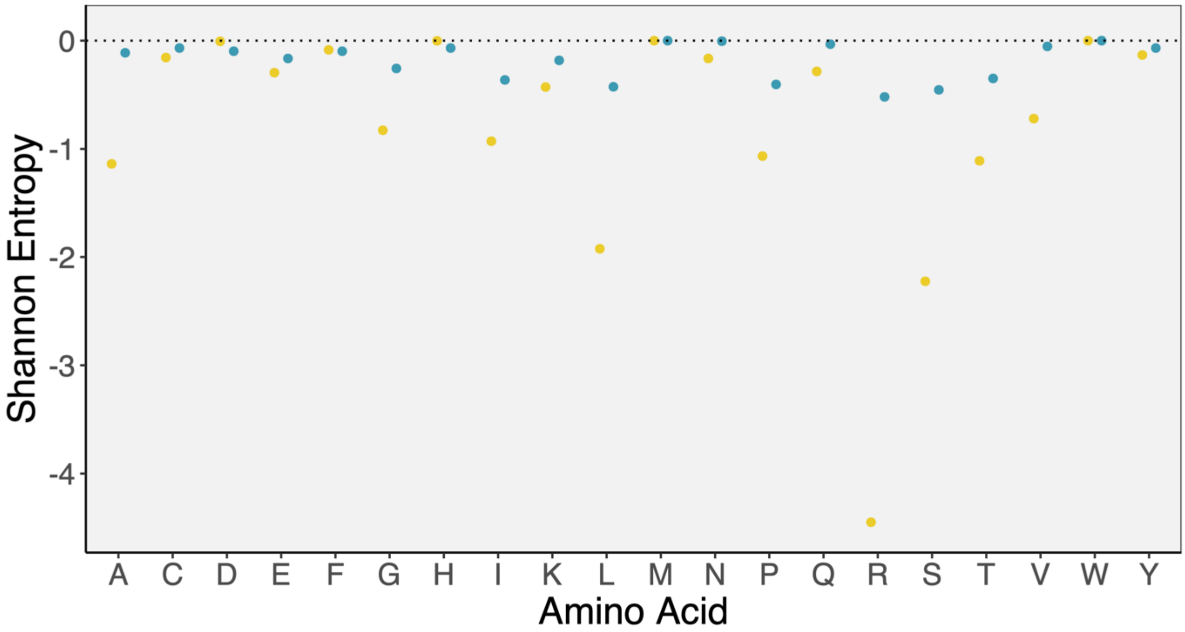

Supplementary Figure S1.2: Shannon entropy for cooperative (blue) and highly-expressed (yellow) genes for each amino acid.

Cooperative genes also have higher GC3 (GC content at third positions) than our set of highly expressed genes. [0.407 in cooperative compared to 0.322 in ribosomal] which may cause them to exhibit variable expression. At first glace, this finding doesn't fit with our hypothesis that cooperative genes are under kin (relaxed) selection, because high GC content is often indicative of highly expressed genes under strong selection (1). However, preferred codons in *B. subtilis* are AT biased (7). Our finding that cooperative genes have high GC content is therefore consistent with relaxed selection because cooperative are less likely to use preferred codons.

The effective number of codons is also substantially higher in cooperative genes than in our set of highly expressed genes (55.1 compared to 43.4), further demonstrating how cooperative genes are using more codons, and therefore less likely to use preferred codons. We also conducted chi-squared tests to determine for each codon whether it was used significantly more than expected (from random chance) in cooperative genes than highly expressed genes, and found that only 14 codons are used significantly more often than expected by chance in cooperative genes, compared to 22 codons that are used significantly more often than expected for highly-expressed genes.

Overall, there is some evidence that decreased usage of preferred codons may explain the increase in synonymous polymorphism. This is the third study to find the counter-intuitive pattern of increased synonymous polymorphism in social genes (*P. aeruginosa* (8) ; *D. discoideum* (9), so more research is needed in this area.

S2: Cooperative vs. background genes

We compared cooperative genes to private genes in the main analysis, and here we also compare to a set of background genes. For the background genes we use those which produce proteins that localise to the cytoplasm, as these are least likely to have cooperative functions. This produces a set of 1832 genes.

Here, we present the results of the post-hoc comparison between cooperative genes and background genes for the main set of molecular population genetic measures

Measure	P-value
Nucleotide polymorphism	0.004*
Non-synonymous polymorphism	0.006*
Synonymous polymorphism	0.056
Ratio of non-synonymous to synonymous polymorphism	0.103
Ratio of non-synonymous to synonymous divergence	0.017*
Non-synonymous divergence	0.004*
Synonymous divergence	0.485
Tajima's D	0.220
Neutrality Index	1.000

We find the same pattern as in the main analysis, with cooperative genes having a signature of higher non-synonymous polymorphism and divergence, without evidence for increased likelihood of positive or balancing selection.

This comparison is less well-controlled than the main analysis, but these results add further weight to our conclusion that signature of selection we observed in quorum sensing - controlled genes is a signature of kin selection.

S3: Balancing selection

A logical explanation for cooperative genes to be more polymorphic than private genes would be that cooperative genes are more likely to be under balancing selection. This could occur if both cheats and cooperators are maintained in a population because the fitness advantage of cheats declines as they become more common (10, 11). It could also occur if multiple greenbeard recognition alleles are maintained (12).

To detect balancing selection from sequence alignment data, we can use several population genetic measures such as Tajima's D and Fu & Li's F* and D* that look at allele frequencies to determine if balancing selection is occurring. Tajima's D looks at the distribution of allele frequencies, whereas Fu & Li's measures look at singletons (rare variants found in only one strain). To interpret these measures, we use statistical tests to determine if each gene is significantly different from the neutral expectation. We then extract the list of genes with significant support, and test if they are overrepresented for social genes using binomial tests.

Tajima's D

We use the beta-distribution test with alpha=0.025 from the Pegas package in R (13, 14) to identify which genes have evidence for balancing selection. The test could be significant either due to balancing selection and a lack of rare alleles ($D>>0$) or a recent selective sweep ($D<<0$), so we exclude significant genes where $D<0$.

We have 242 genes with significant evidence for balancing selection. Only one of those is a cooperative gene, so cooperative genes are not significant overrepresented in those under balancing selection (binomial test, p=0.380). That gene is the aminoglycoside resistance gene aadK.

Fu & Li's D and F**

We use the critical values from (15) for n=100 genes and alpha=0.025, which is 1.53 for D* and 1.73 for F*. The probability of D* or F* being greater than this critical value for by chance is 0.025. Although we have >100 genes, this is likely to be a good approximation as the critical value scales with the natural-log of n.

We have 21 genes with significant evidence for balancing selection from Fu & Li's D*. None of them are cooperative.

Overall, these results support our conclusions that cooperative genes have higher polymorphism due to relaxed selection, rather than balancing selection.

S4: Positive selection

Cooperative genes might show greater divergence than private genes due to being more likely to be under positive or directional selection.

We use several complementary measures to look for signatures of positive selection in our genes. First, we use the Mcdonald-Kreitman (MK) test, which compares the ratio of non-synonymous to synonymous divergence with the ratio of non-synonymous to synonymous polymorphism (16). If there is lots more non-synonymous divergence than non-synonymous polymorphism, then this could indicate positive selection. The logic is that with strong positive selection, advantageous mutations don't spend much time as polymorphisms, and are mainly detected through divergence.

We have 15 genes which have evidence for significant positive selection from the MK test. None of them are cooperative.

Secondly, we use the neutrality index, which uses the same information as the MK test, but rather than just a binary significance test, it uses the full information to make a continuous variable (16). This can be interpreted by comparing averages and distributions of different groups of genes. We log-transformed neutrality index to normalise, meaning that positive values indicate positive selection. There is no difference in neutrality index between cooperative and private genes, and both also don't differ from the background set of genes (Kruskal-Wallis test, chi-squared=0.06, df=2, p=0.974) Supplementary Figure 4.1.

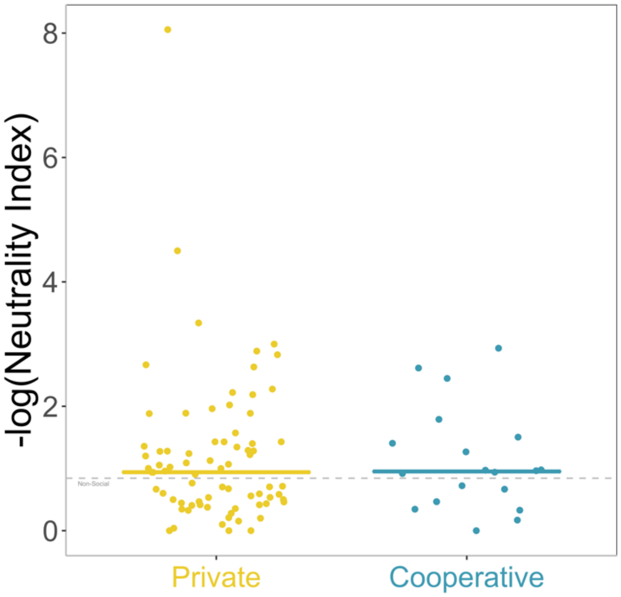

Supplementary Figure 4.1: Log-transformed neutrality index of private and cooperative genes. The dashed line shows the median for background genes.

We also use the direction of selection statistic, which again uses the same information as the MK test to make a continuous variable, with positive values indicating positive selection. Whilst there is a slight trend for cooperative genes to have a higher direction of selection statistic than private genes, this is not significant (Kruskal-Wallis test, chi-squared=0.09, df=2, p=0.955) Supplementary Figure 4.2.

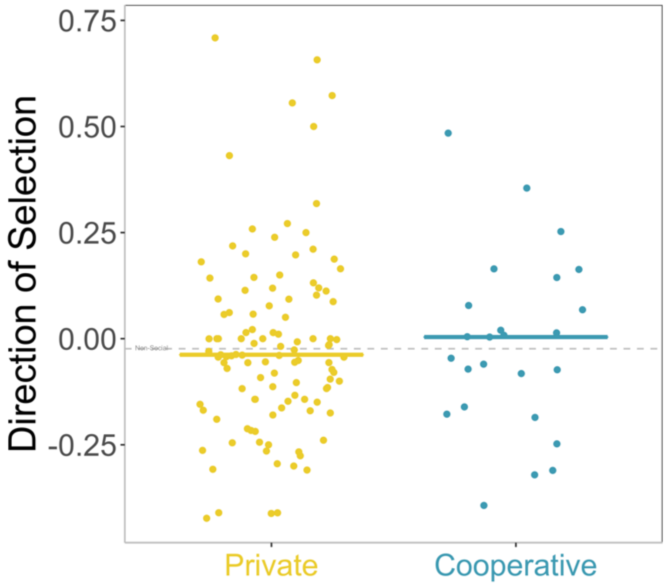

Supplementary Figure 4.2: Direction of selection statistic for private and cooperative genes. The dashed line shows the median for background genes.

Overall, these findings support our conclusion that cooperative genes are not more divergent than private genes due to being more likely to be under positive selection.

Mcdonald-Kreitman conservative

The normal Mcdonald-Kreitman test uses all synonymous differences as the basis of the neutral expectation. Here, we devised and used a modified form of the test named Mcdonal Kreitman-conservative, which considers only the subset of synonymous differences that are most likely to be neutral. We use the logic that synonymous mutations are most likely to be under selection if they switch between preferred codons and unpreferred codons, as codon usage is known to be important in the function and expression of a gene (5). We therefore define a 'conservative' difference as once that either connects two preferred codons, or connects two un-preferred codons. This is inspired by a similar modification to Dn/Ds analysis devised by Zhou *et al* (17).

We used data on preferred codons in *B. subtilis* from Shields and Sharp (1987), which is stored in the R package seqinR (18). We used the R package VariantAnnotation (19) to extract the codon used in each mutation, and a custom script to categorise synonymous mutations as either conservative or not-conservative.

We find that nine of the QS-controlled genes have evidence of significant positive selection, none of which are cooperative.

We also used our method to calculate 'conservative' measures of the Neutrality Index and Direction of Selection statistic, which use information from the Mcdonald Kreitman test. We find no difference between cooperative and private genes for either of these measures (Neutrality Index Wilcoxon rank sum test W=518, p=0.570 Supplementary Figure S4.3; Direction of selection Wilcoxon rank sum test W=1068, p=0.563 Supplementary Figure S4.4).

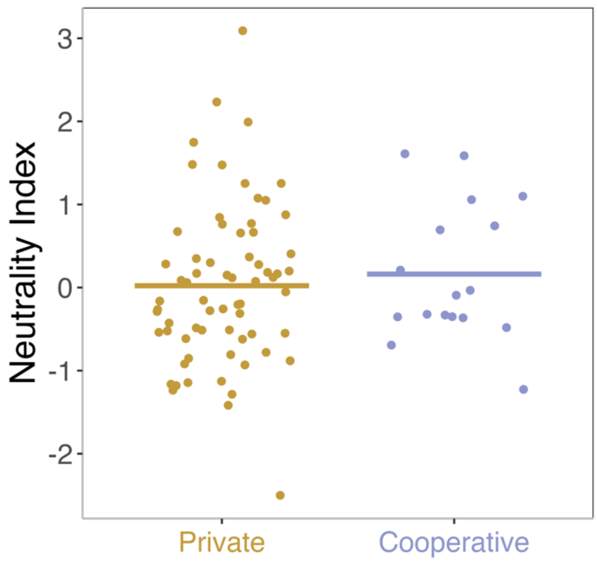

Supplementary Figure 4.3: Log-transformed neutrality index of private and cooperative genes. The dashed line shows the median for background genes.

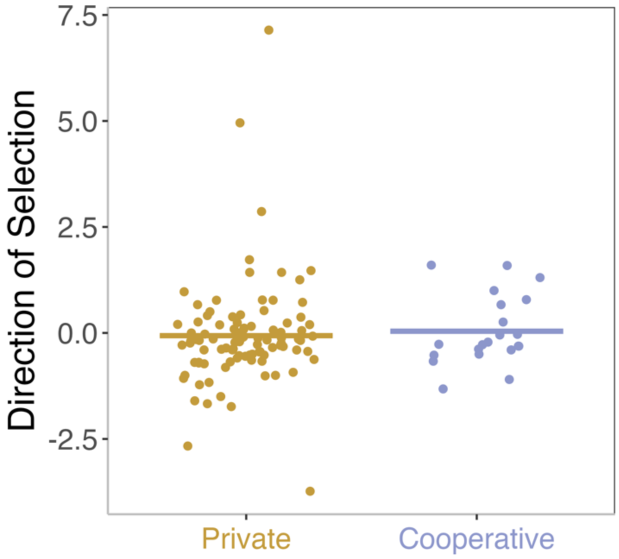

Supplementary Figure 4.4: Direction of selection statistic for private and cooperative genes. The dashed line shows the median for background genes.

S5: Alternative explanations

Gene length

It is well-known that gene length can affect molecular population genetic parameters such as polymorphism. Even though we calculate all measures per site (considering gene length), we also check here that our results aren't an artefact of any differences in gene length between cooperative and private genes.

Cooperative genes are on average 19% longer than private genes (965 base-pairs compared to 812).

We conduct a small analysis where we remove the smallest 25% of genes from our analysis, which is those genes which are <450 base-pairs long.

Cooperative genes are still significantly more polymorphic and divergent than private genes using this reduced dataset (Kruskal-Wallis test, chi-squared=8.16, df=2, p=0.017. Dunn Test p=0.01)

Horizontal gene transfer / pangenome

We focused our analysis on chromosomal genes, because frequent horizontal gene transfer can make drawing conclusion from molecular population genetic parameters more challenging. We check if cooperative genes are more likely to be horizontally transferred by checking if they are overrepresented in the accessory genome compared to the core genome.

B. subtilis has an open pangenome, meaning that each additional strain sequenced adds many new genes. This is usually indicative of a species living in multiple or variable environments, which we know is true for this species. *B. subtilis* is also naturally competent, meaning they can take-up DNA from the environment, and it is thought that the open pangenome occurs because rare genes are acquired in this way from closely related species (20).

We use the panX database (pangenome.org) to assign genes as core or accessory genome based on 80 *B. subtilis* genomes. If we count core genes as those present in 90% of genomes, then 23.2% of the genes in our reference strain are in the *B. subtilis* accessory genome, and 76.8% are in the core genome.

10 out of 53 cooperative genes are in the accessory genome, which is 18.9%. Cooperative genes are therefore not overrepresented in the accessory genome (binomal test p=0.622).

We conclude that cooperative genes are not more likely to be transferred horizontally. This makes sense in light of recent work, which has shown that cooperative genes are not more likely to be carried on plasmids compared to chromosomes across bacteria, including in *B. subtilis* (21). Theory also tells us that cooperation is not favoured by horizontal gene transfer, and so we don't expect cooperative genes to be overrepresented on plasmids or other mobile genetic elements (22).

Power analysis

We conducted a power analysis to see what would happen if we had fewer strains in our population genetic analysis.

We took advantage of the vcf-tools software, which allowed us to randomly remove strains from our analysis. This enabled us to conduct a basic analysis on polymorphism for groups of N strains (N = 4,6,8,10,12,14,16,18,20,22,24,26,28), with 22 iterations of each number of strains

Within any analysis, we focussed on the 3570 genes which are present in all strains, and calculated mean and median polymorphism (average pairwise polymorphism, relative to gene length) (Supplementary Figure S5.1).

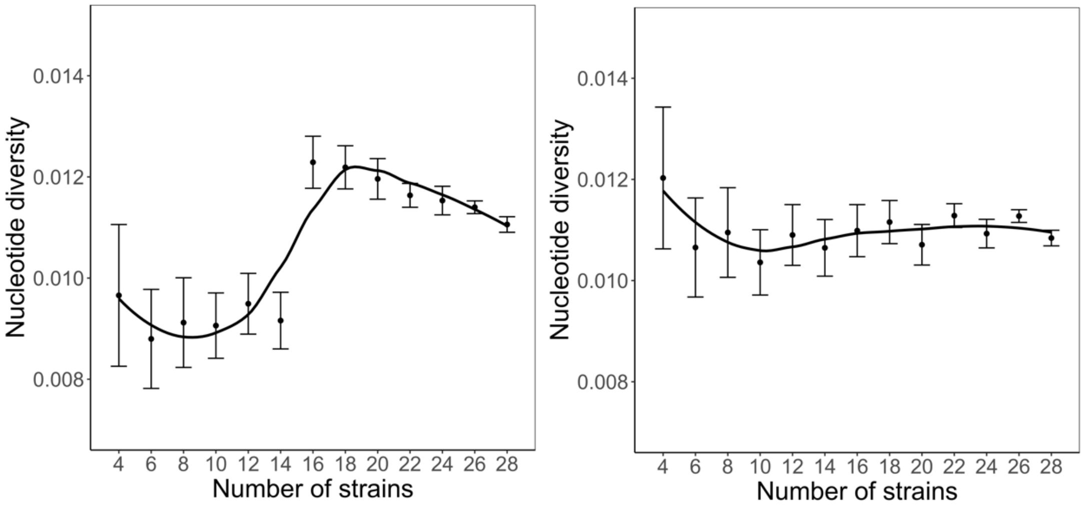

Supplementary Figure S5.1: (A) Median polymorphism, (B) Mean polymorphism as the number of strains uses in the analysis varies. The line is a loess regression fit

The graph on the left shows median polymorphism, which shows a threshold effect once we get to 16 strains, and also much smaller error bars. This shows that a smaller number of strains will miss a lot of the diversity between strains.

The graph on the right shows mean polymorphism. The main pattern is that the standard error in mean polymorphism declines substantially as the number of strains increase. This means that the likelihood of getting a good estimate of the population mean increases as the number of strain increases, which makes logical sense.

The fact that mean polymorphism doesn't change much, but median does, implies that the distribution has changed. The overall conclusion is that the number of strains we have included is likely appropriate to capture the true variation in the population

S6: Division of labour

We conducted an analysis to see if the division of labour in the production of public goods in *B. subtilis* could explain the signatures of selection that we observe. For this, we look at the subset of 40 genes which are controlled by Spo0A, which is active only in a subset of cells (23). Depending on whether Spo0A is on or off, the expression of extracellular polysaccharides and amyloid protein fibres is either repressed or not (24, 25).

Spo0A controlled genes have lower median polymorphism than other quorum sensing -controlled genes, whether measured as overall polymorphism (0.0045 vs. 0.0101), non-synonymous polymorphism (0.0015 vs. 0.0037), or synonymous polymorphism (0.016 vs. 0.021).

Spo0A controlled genes also have lower non-synonymous divergence (0.013 vs. 0.023) and lower ratio between non-synonymous and synonymous divergence (0.049 vs. 0.097), although they have slightly higher synonymous divergence (0.286 vs. 0.266).

The direction of selection statistic is very similar between the two groups (-0.044 vs. -0.037).

This pattern is the opposite to what we would expect if the division of labour was responsible for the signature of selection, rather than sociality *per se*. If the lower conditional expression was having a large effect, then we would expect these genes to have higher polymorphism than the background set of quorum sensing-controlled genes.

Further, EPS and TasA genes stand out within this class of genes as having high polymorphism, implying that the social effect is important in causing the signature of selection that we observe.

S7: Competence

The reference strain NCIB 3610 has a plasmid-encoded gene ComI, which interferes with the competence machinery (Konkol 2013). We know that there is variation in natural competence in our strains, as they were all screened for competency by (26). 18 out of the 31 strains we used are genetically competent.

Some of the competence genes (*comGF, comGE, comGG*) have extremely high polymorphism. Here, we conduct a small analysis where we restrict our analysis to only competent strains, to see if this polymorphism is caused by disuse in non-competent strains. We find that the competence operon *comG* is still amongst the most polymorphic even when we are only looking at competent strains.

Furthermore, cooperative genes are still significantly more polymorphic than private genes in the competent strains (Kruskal-Wallis test, chi-squared=14.7, df=2, $p<0.0001$).

Overall, we conclude that variation in natural competence isn't responsible for the difference between cooperative and private genes that we observe.

S8: Estimation of relatedness

According to theory, the degree to which selection is relaxed in cooperative genes relative to private genes is inversely proportional to relatedness. This result emerges from a simple population genetics model, which shows that a slightly deleterious allele with a cooperative effect on fitness will reach equilibrium frequency inversely proportional to the relationship between the actor and recipient (r) (27). If we assume weak selection and a large population (and ignore higher-order terms), we can directly map this prediction to relative levels of nucleotide polymorphism between alleles with cooperative and private effects on fitness.

As we noted in our previous work on *P. aeruginosa,* we have to make further assumptions that our set of cooperative genes experience the same average strength of selection and distribution of fitness effects as private genes, but this approach has the advantage of many experimental attempts to estimate relatedness for social interactions in that we don't need to know the scale at which interactions take place, or the relative weighting of environments in which traits are more or less social.

Cooperative genes have a median polymorphism of 0.0122, and private genes have median polymorphism of 0.0096. This leads to a calculation of relatedness as r=0.79.

We note that this measure might vary depending on how you define the population, which is tricky in bacteria due to horizontal gene transfer and other complications (28). In *B. subtilis*, for example, we know that strains from the same plant root or gram of soil can vary in their production of public goods, and don't always group together phylogenies (29–31).

S9: Correlation in gene expression for secondary comparisons

Here, we use the dataset from Futo *et al*. 2020 to test if the genes we used in the secondary comparisons also tend to be co-expressed. For each set of genes we first calculate the mean pairwise correlation between all possible pairs of genes. We then use a bootstrap approach of randomly sampling 10,000 other gene sets of the same size, to see if the gene set has higher correlated expression than expected by chance.

For all gene sets, we find that the correlation is greater than >95% of randomly sampled gene-sets (Supplementary Table S9.1)

Supplementary Table S9.1: Results from pairwise correlation in gene expression of a gene set, and bootstrap iterations of randomly sampled gene sets of the same size

Gene set	Correlation for gene set	Mean correlation for 10,000 iterations	Percentile of gene set
Iron scavenging	0.880	0.329	100
Antibiotic resistance	0.429	0.306	95.7
Proteases	0.397	0.293	95.3
Toxins	0.534	0.305	99.8
Antimicrobials	0.640	0.294	100

S10: Positive and balancing selection in other cooperative traits

We checked whether the other cooperative traits that we examined differ in signatures of positive and balancing selection, as this could cloud our conclusion on the nature of selection. We use Tajima's D to detect balancing selection, and neutrality index to detect positive selection.

Cooperative genes don't differ in balancing selection, whether we use all genes from the six comparisons (ANOVA $F_{2,121} = 7.50, p < 0.001$; Games Howell Test p=0.39), just the five secondary comparisons (ANOVA $F_{2,44} = 4.72, p = 0.14$; Games Howell Test p=0.33), or consider each gene as a data point (Wilcoxon signed-rank test V=7, p=0.563).

Cooperative genes also don't differ in positive selection, whether we use all genes from the six comparisons (Kruskal-Wallis $X^2(2) = 2.11, p = 0.35, \text{Dunn Test p} = 0.70$), just the five secondary comparisons (Kruskal-Wallis $X^2(2) = 5.37, p = 0.07, \text{Dunn Test p} = 0.98$), or consider each gene as a data point (Wilcoxon signed-rank test V=7, p=0.563).

Overall, we can conclude that the signature of selection we see across the five other cooperative traits are most consistent with kin selection causing the effective relaxation of selection

S11: Correlations in gene expression of QS-controlled genes

To test the robustness of our result that quorum sensing-controlled genes tend to be expressed at the same time, we repeated our analysis using the dataset from Pisithkul (32).

Whilst Futo *et al*. measured gene expression in a solid-air interface biofilm over two months (N=11 timepoints), Pisithkul *et al*. used a liquid-air interface, and measured gene expression over 24 hours (N=7 timepoints), representing the initial stages of biofilm growth.

Pisithkul *et al*. provided their data in the form of reads per kilobase of transcript per million mapped reads. For each gene, we normalised the data with the following steps;

1) We calculated median expression across the four replicates of each timepoint
2) We then divided each measure by the median expression at timepoint one (eight hours). This allows for better comparison between genes
3) We then used a log2 transformation to normalize the resulting relative expression measures

The average correlation for the N=160 quorum sensing controlled genes that we were able to match to the data was 0.411. The average correlation for N=10,000 randomly sampled gene sets of the same size (N=160) was 0.385. Quorum sensing-controlled genes have a higher correlation than 98.5% of the randomly sampled gene sets.

S12: Cooperative operons

The cooperative traits that we analyse form four operons:
Surfactin (*srfAA-srfAB-srfAC-srfAD-srfT*); Extracellular polysaccharides (*epsA-epsB-epsC-epsD-epsE-epsF-epsG-epsH-epsI-epsJ-epsK-epsL-epsM-epsN-epsO*); Biofilm matrix proteins (*tapA-sipW-tasA*); Biofilm surface layer proteins (*bslA-bslB*).

There may be epistatic effects that shape whether selection can act independently on each gene. For example, a mutation that alters the function of one *eps* gene may impact selection on the other *eps* genes. We therefore check the robustness of our results by combining genes in the same operon into one data point. This gives us n=4 for cooperative traits, and n=136 for private traits, which we also combined into operons (Table S12). Our results are quantitatively the same, showing that epistatic interactions are not driving the trends we see in the data (Supplementary Figures S12.1-S12.3).

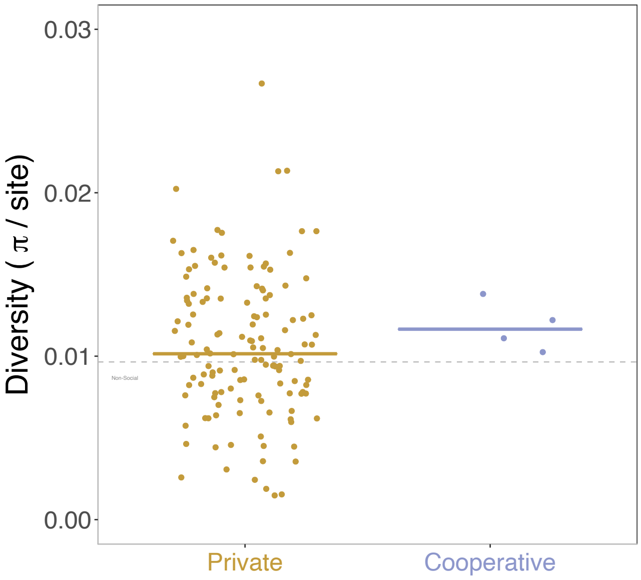

Supplementary Figure S12.1: Nucleotide diversity for private (gold) and cooperative (blue) operons.

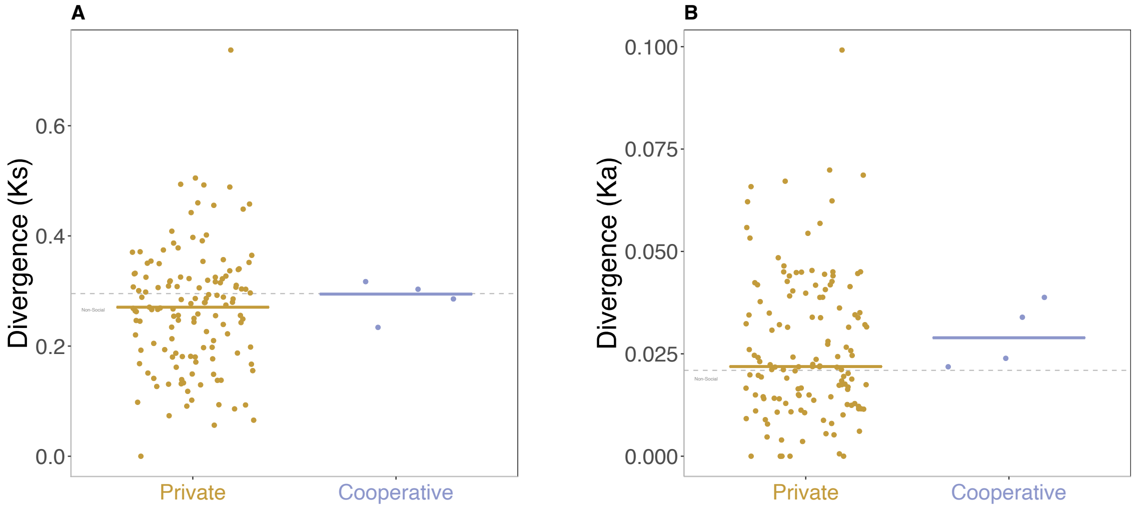

Supplementary Figure S12.2: Divergence at synonymous (A) and non-synonymous (B) sites for private (gold) and cooperative (blue) operons.

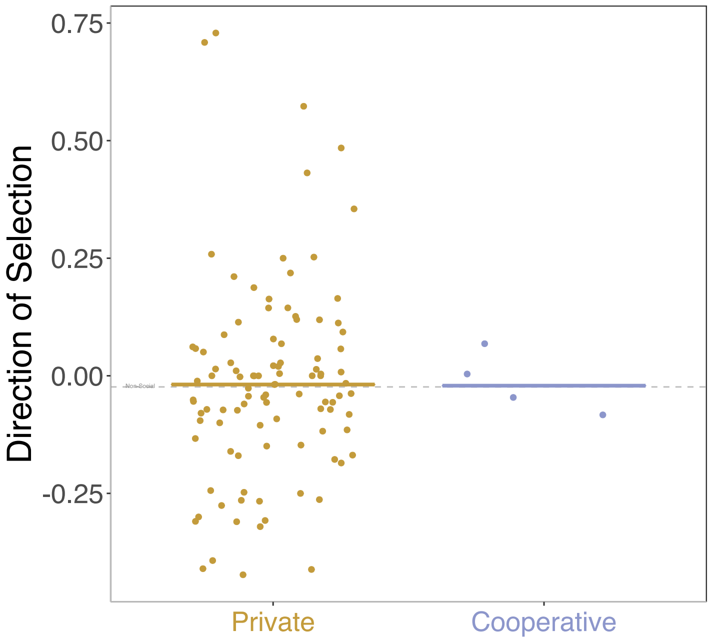

Supplementary Figure S12.3: Direction of Selection statistic for private (gold) and cooperative (blue) operons

**Table S12: Operons for private traits controlled by quorum-sensing**
accA-accD
bacF-bacE-bacD-bacC-bacB-bacA
essA-yueB-essC-essB-yukD-yukE
fapR-fabD-fabG
pgsC-pgsB-edmS
pksA-pksD-pksE-pksF-pksG-pksH-pksI-pksJ-pksL-pksM-pksN-pksR
pnbA-slrR
rapC-phrC
rapF-phrF
sigF-spoIIAB-spoIIAA
xynC-xynD
ydaK-ydaL
yfjF-yfjE-yfjC-yfjB-yfjA
yjhA-yjhB
yppE-yppD
yraI-yraJ
yvcA-yvcB
yvdA-yvdB
yvkA-yvkB-yvkC
ywqH-ywqI-rttN
yxiA-yxiB-yxiC

S13: Essential genes

There are 258 essential genes in *B. subtilis*, as listed on *SubtiWiki* (33). All of these genes are private. It may be hypothesised that polymorphism and divergence will be lower in genes that are essential for an organisms survival, due to the obvious costs of mutation. To check that the signature of selection we observed in cooperative genes wasn't due to differences in gene essentially, we conducted a small analysis where we removed the quorum-sensing controlled genes that are listed as essential. The gene genes in question are: *accA, accD, fabD, fabF, fabG, pgsA, plsC, plsX,* all of which are private genes.

Removing these genes had no effect on the difference in polymorphism between cooperative and private genes (Supplementary Figure S13.1).

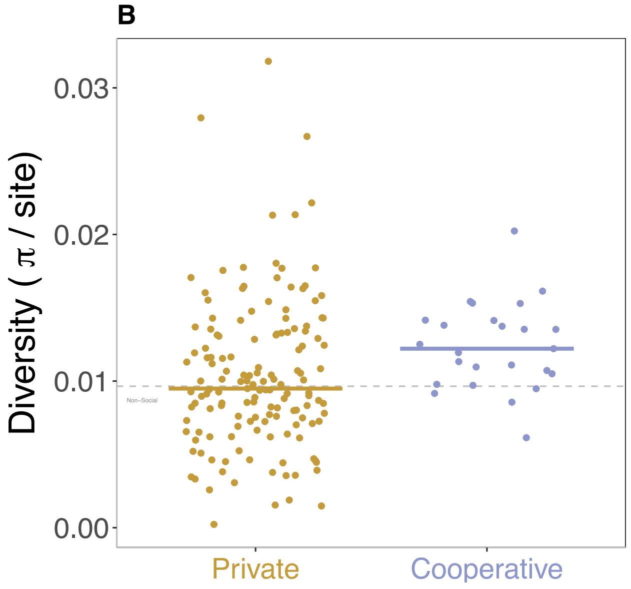

Supplementary Figure S13.1: Nucleotide diversity for private (gold) and cooperative (blue) genes, including only genes that aren't essential.

S14: Gene expression level

In the main text we analysed genes that are switched on and off together (co-expressed). Here, we look at the maximum expression level of genes, and test for any difference between cooperative and private genes. Genes that are highly expressed tend to be shorter, and show higher codon bias (34).

We used the dataset from Pisithkul *et al.* (2019), which provides gene expression in the form of reads per kilobase of transcript per million reads (RPKM). For each gene at each timepoint, we calculated the median across replicates. We then defined the maximum expression of that gene as the largest of those median values. We log-transformed the data for normality. We found that cooperative genes don't have different expression levels compared to private genes (t-test t=0.0977, df=27, p=0.923) (Supplementary Figure S14.1).

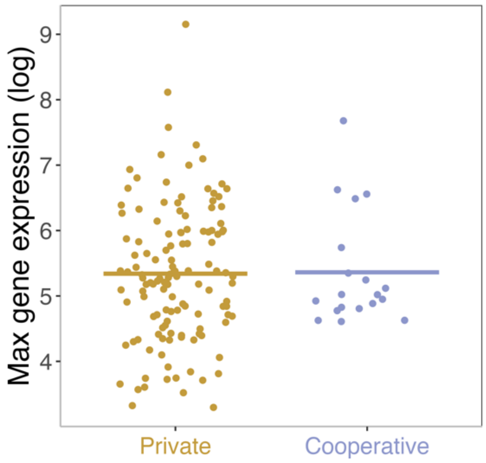

Supplementary Figure S14.1: Plot showing the maximum expression level of cooperative genes compared to private genes. Private genes are shown in gold, and cooperative genes are in blue. Each point is a gene, and the bar represents the mean.

We also tested for any correlation between expression levels and nucleotide polymorphism. Higher expressed genes have lower nucleotide polymorphism (linear model $F_{(1,145)}$=10.22, p<0.01), but the correlation is weak (r-squared = 0.06) (Supplementary Figure S14.2).

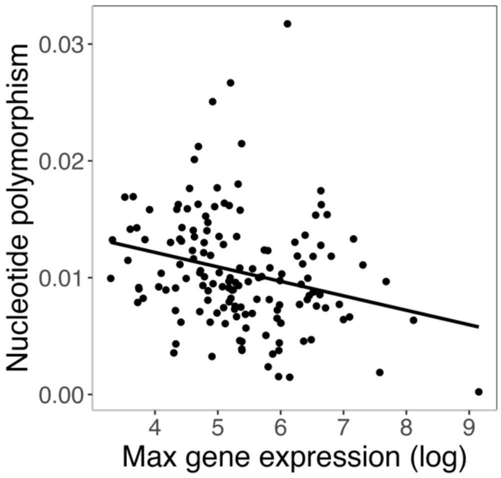

Supplementary Figure S14.2: Correlation between the maximum expression level of a gene and its nucleotide polymorphism.

S15: Conditional expression

Whilst our main analysis on genes controlled by quorum sensing controls for conditional expression in general, there is an 'extra' level of conditional expression caused by cooperative genes that are both QS-controlled and expressed in only a subset of cells. Here, we investigate if this extra level of conditional expression in some cooperative genes could explain the signatures of selection we observe.

We measured nucleotide polymorphism in five sets of genes, listed below. This then allowed us to make different comparisons between genes that differ in levels of conditionality (non-conditional - conditional - extra-conditional) or sociality (private – cooperative).

(1) Essential genes (non-conditional, non-social)
(2) private QS genes (conditional, non-social)
(3) Spo0A-controlled genes that are QS-controlled (super-conditional, non-social)
(4) epsa-O & tapA-sipW-tasA (super conditional, social)
(5) other cooperative QS genes (conditional, social)

Tests of the effect of conditionality

The difference between (1) and (2) and (3) is conditionality. This is a comparison between non-conditional essential genes, private QS genes, and the extra-conditional Spo0A-controlled genes. The level of conditionality had a significant effect (ANOVA $F_{2,376}$=14.55, $p<10^{-6}$). Private QS has significantly higher polymorphism that essential genes (Tukey HSD $p<0.00001$). However, genes that are QS-regulated and Spo0A regulated (super-conditional) don't have higher polymorphism than the essential genes ($p=0.942$) (Figure S15.1).

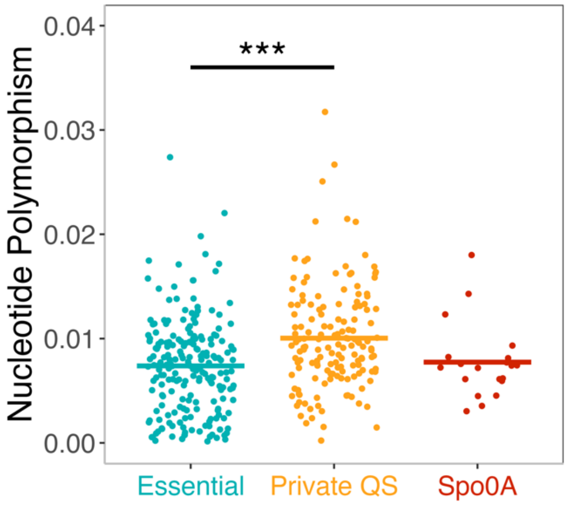

Figure S15.1: Nucleotide polymorphism for three groups of genes that differ in levels of conditionality from low (left) to high (right). Each point is a gene, with the bar representing the mean for that group. The black bar and * represents the significance of pairwise comparisons from a Tukey's HSD post-hoc test.

The difference between (4) and (5) is conditionality. This is a comparison between cooperative extracellular matrix genes known to be expressed only in a subset of cells (epsA-O & tapA-

sipW-tasA) to other cooperative QS genes. The level of conditionality had no effect (t-test t=0.0438, df=13, p=0.966) (Figure S15.2).

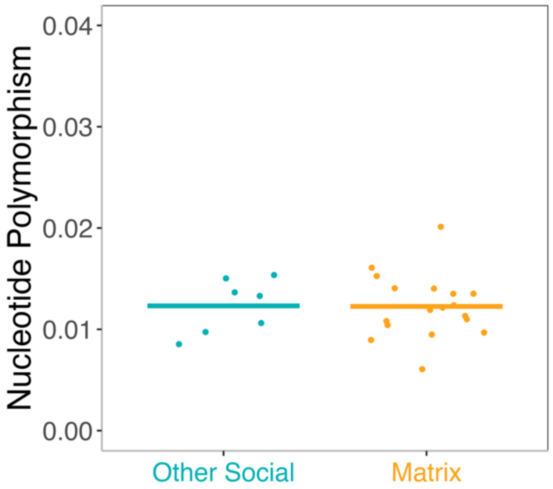

Figure S15.2: Nucleotide polymorphism for two groups of genes that differ in levels of conditionality from low (left) to high (right). Each point is a gene, with the bar representing the mean for that group.

Tests of the effect of sociality

The difference between (3) and (4) is sociality. This is a comparison between Spo0A-controlled QS genes (extra-conditional private genes) with extracellular matrix genes (extra-conditional cooperative genes). The cooperative genes had a significantly higher polymorphism than private genes (t-test t=-4.150, df=36, p<0.001) (Figure S15.3).

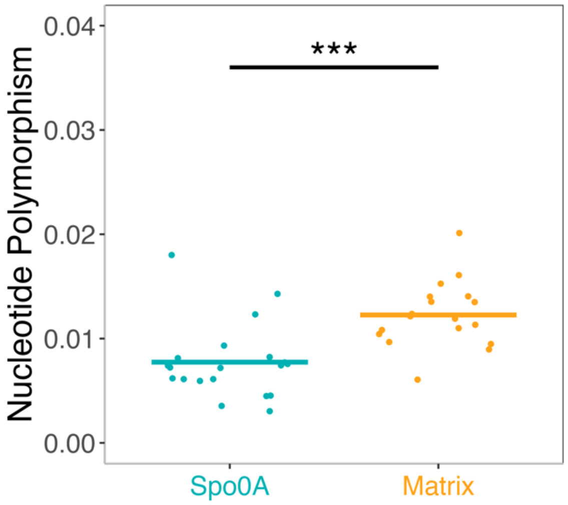

Figure S15.3: Nucleotide polymorphism for two groups of genes that differ in levels of sociality from private (left) to cooperative (right). Each point is a gene, with the bar representing the mean for that group. The black bar and * represents the significance of pairwise comparisons using a t-test.

A combined model

To test the effects of sociality and conditionality, we also made a linear model that combined the data from these groups. The overall model was significant (ANOVA $F_{3,400}$=15.45, $p<10^{-8}$). We found that higher conditionality and higher sociality both led to significantly higher polymorphism, as predicted by theory (conditionality $p<10^{-6}$; sociality $p<10^{-4}$). However, sociality explained almost twice the amount of the variation in polymorphism compared to conditionality (6.76% vs 3.83%). The jump from non-conditional to QS-controlled seems to be important, but the extra conditionality of some QS-controlled genes doesn't have an effect on polymorphism. This gives us confidence that we are able to separate the two effects.

Supplementary Figures

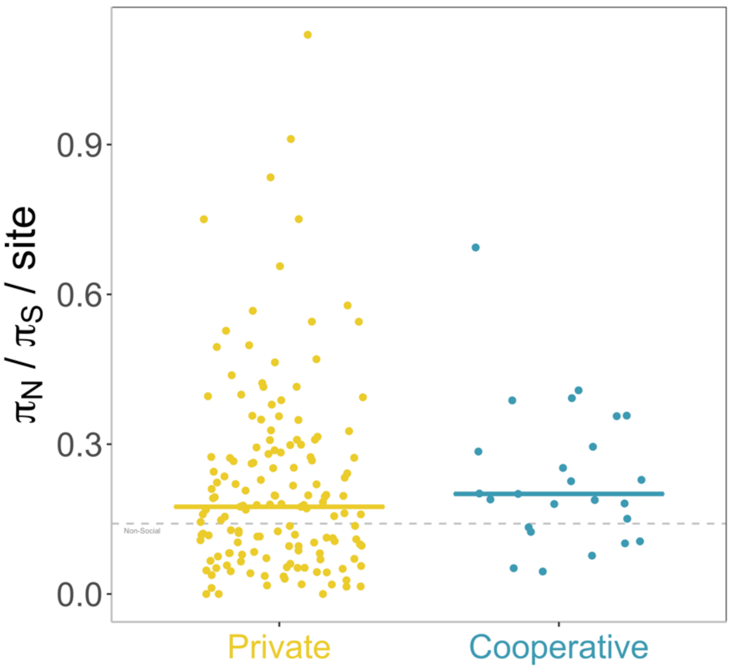

Supplementary Figure 1: Ratio between non-synonymous and synonymous nucleotide diversity per site for private (yellow) and cooperative (blue) genes controlled by quorum sensing. Each point is a gene, and the horizontal line shows the median for each group. The grey line shows the median for background private genes across the genome.

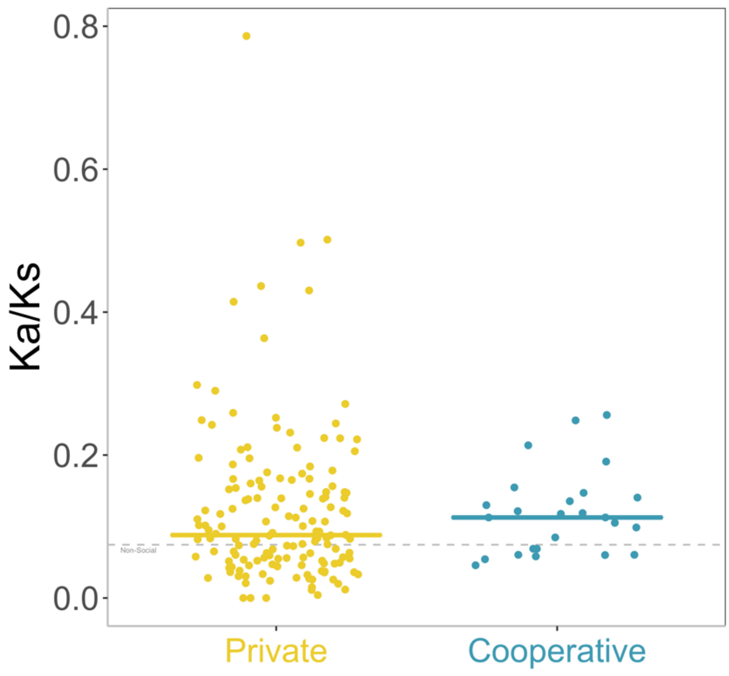

Supplementary Figure 2: Ratio between non-synonymous and synonymous divergence for private (yellow) and cooperative (blue) genes controlled by quorum sensing. Divergence is measured by rates of protein evolution, e.g. number of synonymous substitutions per synonymous site for panel B. Each point is a gene, and the horizontal line shows the median for each group. The grey line shows the median for background private genes across the genome.

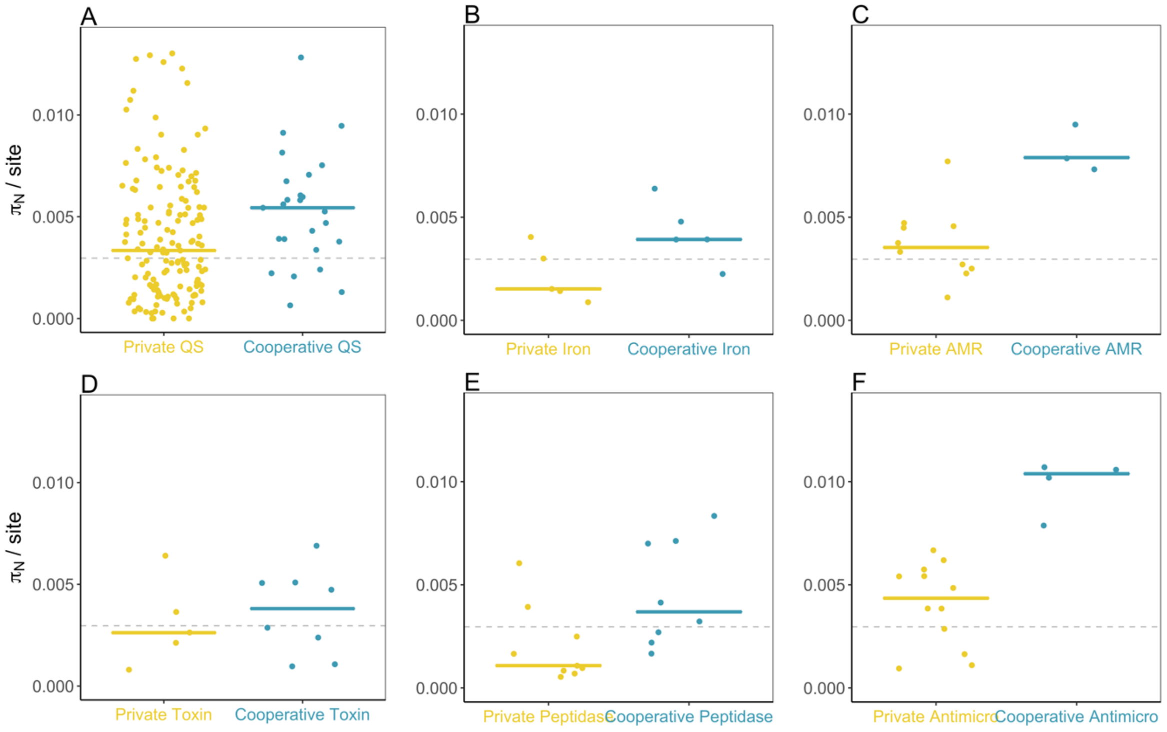

Supplementary Figure 3: Private (yellow) vs. cooperative (blue) non-synonymous polymorphism in genes for six traits. Panel A shows the quorum sensing -controlled genes used in the main analysis. Panels B-F show the secondary comparisons

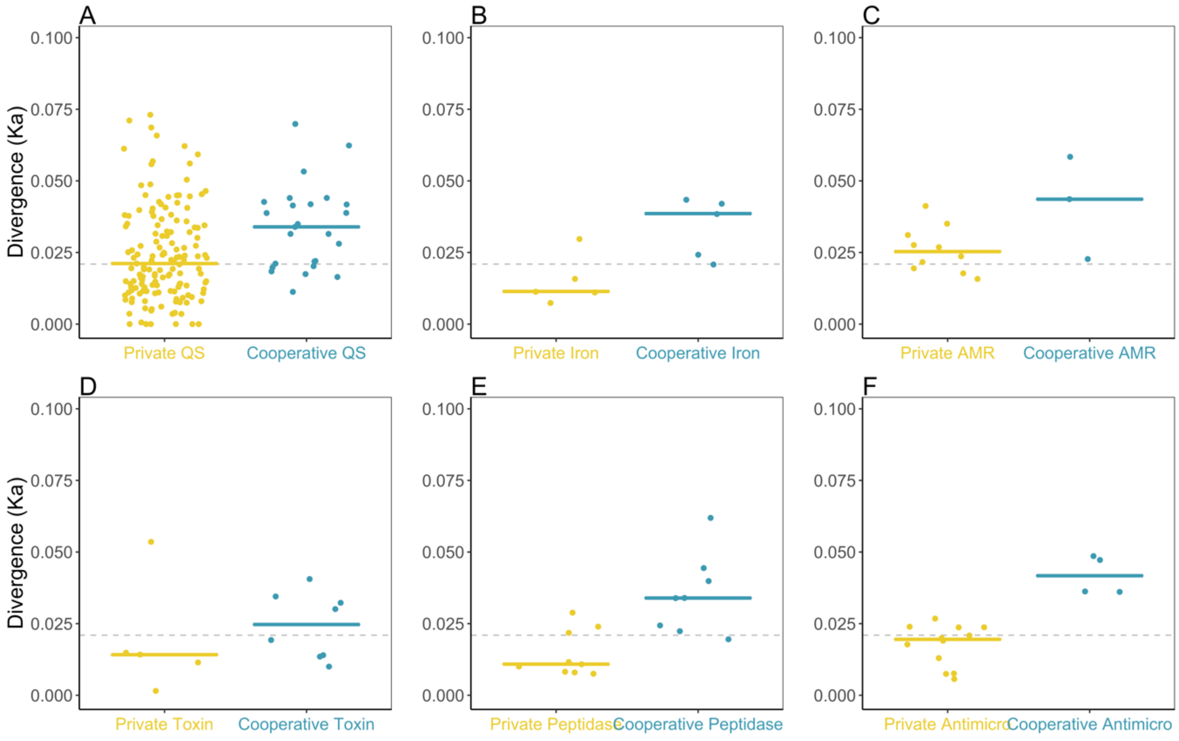

Supplementary Figure 4: Private (yellow) vs. cooperative (blue) non-synonymous divergence in genes for six traits. Panel A shows the quorum sensing -controlled genes used in the main analysis. Panels B-F show the secondary comparisons

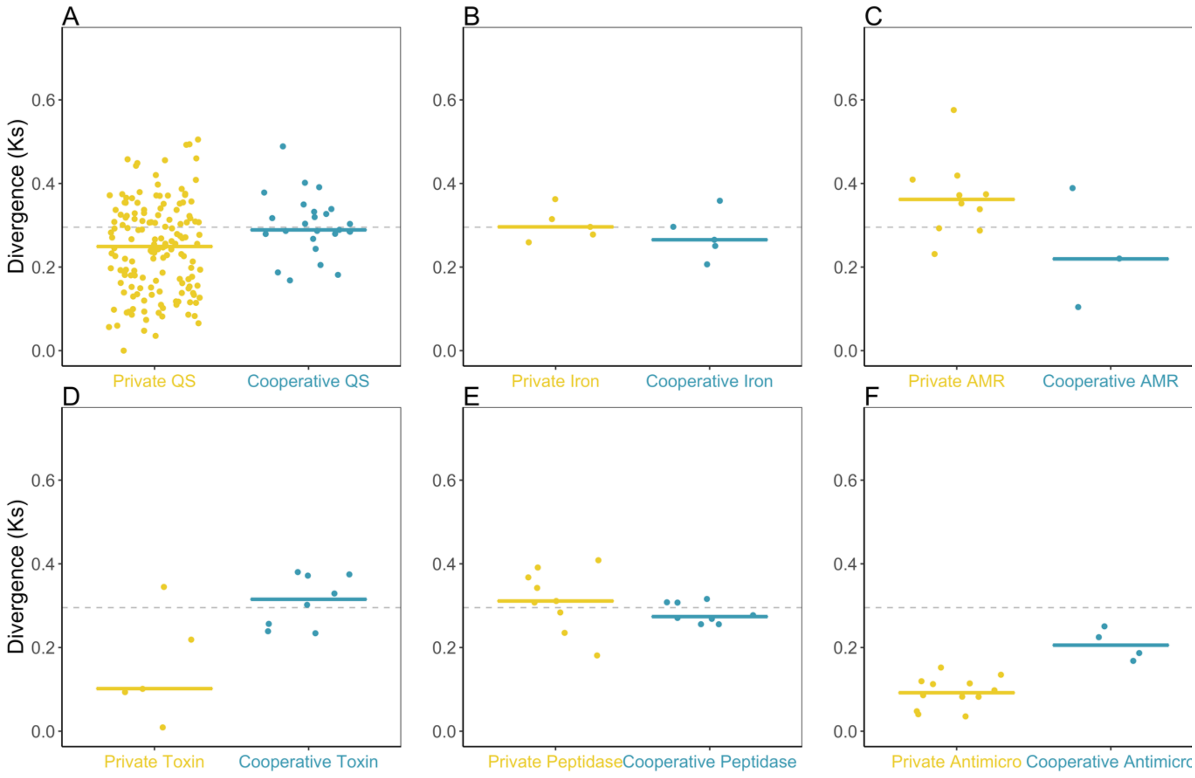

Supplementary Figure 6: Private (yellow) vs. cooperative (blue) synonymous divergence in genes for six traits. Panel A shows the quorum sensing -controlled genes used in the main analysis. Panels B-F show the secondary comparisons

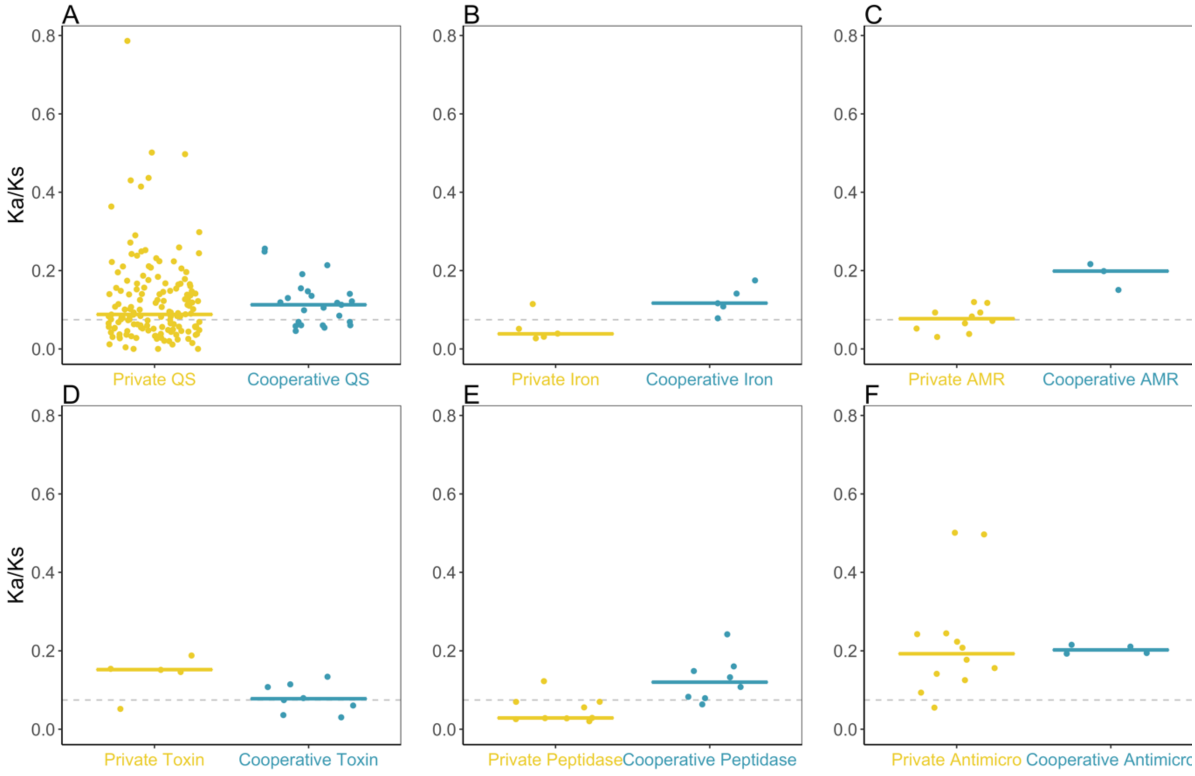

Supplementary Figure 7: Private (yellow) vs. cooperative (blue) ratio between non-synonymous and synonymous divergence in genes for six traits. Panel A shows the

quorum sensing-controlled genes used in the main analysis. Panels B-F show the secondary comparisons

Supplementary Tables

Supplementary Table 1: List of strains used

Strain ID	Location	Assembly
NRS6096	Tayport (UK) garden soil	GCA_905311035.1
NRS6099	Tayport (UK) garden soil	GCA_905310985.1
NRS6103	Tayport (UK) community garden soil	GCA_905310995.1
NRS6105	Tayport (UK) community garden soil	GCA_905311425.1
NRS6107	Tayport (UK) garden soil	GCA_905311405.1
NRS6108	Tayport (UK) garden soil	GCA_905311395.1
NRS6110	Tayport (UK) vegetable plot	GCA_905311415.1
NRS6111	Tayport (UK) vegetable plot	GCA_905311375.1
NRS6116	Tayport (UK) garden soil	GCA_905311385.1
NRS6118	Tayport (UK) potato patch	GCA_905311435.1
NRS6120	Tayport (UK) potato patch	GCA_905312035.1
NRS6121	Tayport (UK) shrub bed	GCA_905315035.1
NRS6127	Tayport (UK) garden soil	GCA_905315045.1
NRS6128	Tayport (UK) garden soil	GCA_905315055.1
NRS6131	Tayport (UK) worm bin	GCA_905315025.1
NRS6134	Tayport (UK) vegetable patch	GCA_905315395.1
NRS6137	Tayport (UK) community garden soil	GCA_905315385.1
NRS6141	Tayport (UK) garden soil	GCA_905315375.1
NRS6145	Tayport (UK) garden soil	GCA_905315685.1
NRS6148	Tayport (UK) garden soil	GCA_905315695.1
NRS6153	Tayport (UK) garden soil	GCA_905315705.1
NRS6160	Tayport (UK) garden soil	GCA_905315715.1
NRS6167	Tayport (UK) soil from planter	GCA_905316385.1
NRS6181	Tayport (UK) garden soil	GCA_905318255.1
NRS6183	Lochee (UK) garden soil	GCA_905319155.1
NRS6186	Newport (UK) garden soil	GCA_905319135.1
NRS6190	Tayport (UK) garden soil	GCA_905319565.1
NRS6194	Tayport (UK) garden soil	GCA_905319555.1
NRS6202	Kirriemuir (UK) garden soil	GCA_905319535.1
NRS6205	Kirriemuir (UK) garden soil	GCA_905319825.1
NRS6206	Kirriemuir (UK) garden soil	GCA_905319815.1

Supplementary Table 2: List of social genes

Gene	Function	Reference
**bslA**	biofilm-surface layer protein BslA	(35, 36)
**bslB**	biofilm-surface layer protein BslB	(37)
**epsA**	hypothetical protein	(35, 36)
**epsB**	protein tyrosine kinase EpsB	(35, 36)
**epsC**	polysaccharide biosynthesis protein	(35, 36)
**epsD**	glycosyltransferase family 4 protein	(35, 36)
**epsE**	glycosyltransferase EpsE	(35, 36)
**epsF**	glycosyltransferase family 1 protein	(35, 36)
**epsG**	biofilm exopolysaccharide biosynthesis protein EpsG	(35, 36)
**epsH**	glycosyltransferase	(35, 36)
**epsI**	polysaccharide pyruvyl transferase family protein	(35, 36)
**epsJ**	lipoprotein	(35, 36)
**epsK**	cyclic-di-GMP receptor EpsK	(35, 36)
**epsL**	sugar transferase	(35, 36)
**epsM**	acetyltransferase	(35, 36)
**epsN**	aminotransferase class I/II-fold pyridoxal phosphate-dependent enzyme	(35, 36)
**epsO**	polysaccharide pyruvyl transferase family protein	(35, 36)
**sipW**	signal peptidase I	(35, 36)
**srfAA**	surfactin non-ribosomal peptide synthetase SrfAA	(35, 36)
**srfAB**	surfactin non-ribosomal peptide synthetase SrfAB	(35, 36)
**srfAC**	surfactin non-ribosomal peptide synthetase SrfAC	(35, 36)
**srfAD**	surfactin biosynthesis thioesterase SrfAD	(35, 36)
**srfT**	MFS transporter	(35, 36)
**tapA**	amyloid fiber anchoring/assembly protein TapA	(35, 36)
**tasA**	biofilm matrix protein TasA	(35, 36)

Supplementary Table 3: Cooperative and private genes for iron-scavenging via bacillibactin

Gene ID	Name	Function	Sociality	Reference
**IRON SCAVENGING**				
**17270**	dhbF	Bacillibactin biosynthesis	Cooperative	(38, 39)
**17275**	dhbB	Bacillibactin biosynthesis	Cooperative	(38, 39)
**17280**	dhbE	Bacillibactin biosynthesis	Cooperative	(38, 39)
**17285**	dhbC	Bacillibactin biosynthesis	Cooperative	(38, 39)
**17290**	dhbA	Bacillibactin biosynthesis	Cooperative	(38, 39)
**1040**	feuC	Membrane permease	Private	(38, 39)
**1045**	feuB	Membrane permease	Private	(38, 39)
**1050**	feuA	Periplasmic binding protein	Private	(38, 39)
**17785**	yusV [feuV]	ABC transporter	Private	(38, 39)
**17295**	besA	Ferri-bacillibactin esterase	Private	(38, 39)

Supplementary Table 4: Cooperative and private genes for antibiotic resistance

Gene ID	Name	Function	Sociality	Reference

**ANTIBIOTIC RESISTANCE**				
**1295**	blaOXA (ybxI)	Beta-lactamase	Cooperative	(40)
**10225**	bla (penP)	Beta-lactamase	Cooperative	(40)
**14460**	aadk	Aminoglycoside resistance	Cooperative	(41)
**5445**	bmrC	Multidrug ABC transporter	Private	(42)
**5550**	bmrD	Multidrug ABC transporter	Private	(42)
**20835**	cydC	Multidrug ABC transporter	Private	(42)
**20840**	cydD	Multidrug ABC transporter	Private	(42)
**4595**	yfiB	Multidrug ABC transporter	Private	(42)
**4600**	yfiC	Multidrug ABC transporter	Private	(42)
**7900**	yknU	Multidrug ABC transporter	Private	(42)
**7905**	yknV	Multidrug ABC transporter	Private	(42)
**20050**	ywjA	Multidrug ABC transporter	Private	(42)
**18775**	bmrA	Multidrug ABC transporter	Private	(42)
**4830**	ygaD	Multidrug ABC transporter	Private	(42)
**11680**	sunT	Sublancin transporter	Private	(42)

Supplementary Table 5: Cooperative and private genes toxin genes

Gene ID	Name	Function	Sociality	Reference
**TOXINS**				
**20325**	ywfA	Bacilycin biosysnthesis & export	Cooperative	(43)
**20320**	bacA	Bacilycin biosysnthesis & export	Cooperative	(43)
**20315**	bacB	Bacilycin biosysnthesis & export	Cooperative	(43)
**20310**	bacC	Bacilycin biosysnthesis & export	Cooperative	(43)
**20305**	bacD	Bacilycin biosysnthesis & export	Cooperative	(43)
**20300**	bacE	Bacilycin biosysnthesis & export	Cooperative	(43)
**20295**	bacF	Bacilycin biosysnthesis & export	Cooperative	(43)
**20290**	bacG	Bacilycin biosysnthesis & export	Cooperative	(43)
**3890**	yeeF	LXG toxin	Private	(44)
**10360**	yobL	LXG toxin	Private	(44)
**11735**	yokI	LXG toxin	Private	(44)
**1830**	yqcG	LXG toxin	Private	(44)
**19495**	ywqJ (rttN)	LXG toxin	Private	(44)
**21125**	yxiD	LXG toxin	Private	(44)

Supplementary Table 6: Cooperative and private genes protease genes

Gene ID	Name	Function	Sociality	Reference
**PROTEASES**				
**5755**	aprE	Extracellular protease	Cooperative	(45)
**8405**	Bpr	Extracellular protease	Cooperative	(45)
**20660**	Epr	Extracellular protease	Cooperative	(45)
**1375**	Mpr	Extracellular protease	Cooperative	(45)
**6155**	nprB	Extracellular protease	Cooperative	(45)
**8100**	nprE	Extracellular protease	Cooperative	(45)
**20500**	Upr	Extracellular protease	Cooperative	(45)
**5990**	wprA	Extracellular protease	Cooperative	(45)
**9385**	aprX	Intracellular protease	Private	(45)
**7560**	clpE	Intracellular protease	Private	(45)
**18630**	clpP	Intracellular protease	Private	(45)

**8830**	clpQ	Intracellular protease	Private	(45)
**13140**	ispA	Intracellular protease	Private	(45)
**15210**	lonA	Intracellular protease	Private	(45)
**15215**	lonB	Intracellular protease	Private	(45)
**9115**	mlpA	Intracellular protease	Private	(45)
**12000**	ypwA	Intracellular protease	Private	(45)

Supplementary Table 7: Cooperative and private genes for the production of antimicrobials

Gene ID	Name	Function	Sociality	Reference
**PROTEASES**				
**9975**	ppsA	Plipistatin biosynthesis	Cooperative	(46)
**9970**	ppsB	Plipistatin biosynthesis	Cooperative	(46)
**9965**	ppsC	Plipistatin biosynthesis	Cooperative	(46)
**9960**	ppsD	Plipistatin biosynthesis	Cooperative	(46)
**9955**	ppsE	Plipistatin biosynthesis	Cooperative	(46)
**9295**	pksB	Bacillaene biosynthesis	Private	(47)
**9310**	pksD	Bacillaene biosynthesis	Private	(47)
**9315**	pksE	Bacillaene biosynthesis	Private	(47)
**9325**	pksF	Bacillaene biosynthesis	Private	(47)
**9330**	pksG	Bacillaene biosynthesis	Private	(47)
**9335**	pksH	Bacillaene biosynthesis	Private	(47)
**9340**	pksI	Bacillaene biosynthesis	Private	(47)
**9345**	pksJ	Bacillaene biosynthesis	Private	(47)
**9350**	pksL	Bacillaene biosynthesis	Private	(47)
**9355**	pksM	Bacillaene biosynthesis	Private	(47)
**9360**	pksN	Bacillaene biosynthesis	Private	(47)
**9365**	pksR	Bacillaene biosynthesis	Private	(47)
**9370**	pksS	Bacillaene biosynthesis	Private	(47)

Supplementary References

1. F. Hildebrand, A. Meyer, A. Eyre-Walker, Evidence of selection upon genomic GC-content in bacteria. *PLoS Genet.* **6** (2010).
2. P. M. Sharp, E. Bailes, R. J. Grocock, J. F. Peden, R. E. Sockett, Variation in the strength of selected codon usage bias among bacteria. *Nucleic Acids Res.* **33**, 1141–1153 (2005).
3. H. Akashi, T. Gojobori, Metabolic efficiency and amino acid composition in the proteomes of Escherichia coli and Bacillus subtilis. *Proc. Natl. Acad. Sci. U. S. A.* **99**, 3695–3700 (2002).
4. R. Raghavan, Y. D. Kelkar, H. Ochman, A selective force favoring increased G+C content in bacterial genes. *Proc. Natl. Acad. Sci. U. S. A.* **109**, 14504–14507 (2012).
5. E. P. C. Rocha, Codon usage bias from tRNA's point of view: Redundancy, specialization, and efficient decoding for translation optimization. *Genome Res.* **14**, 2279–2286 (2004).
6. Y. Sun, S. G. E. Andersson, SSCU: an R/Bioconductor package for analyzing selective profile in synonymous codon usage (2018).
7. D. . Shields, P. M. Sharp, Synonymous codon usage in Bacillus subtilis reflects both translational selection and mutational biases. *Nucleic Acids Res.* **15**, 8235–8251

(1987).
8. L. J. Belcher, A. E. Dewar, M. Ghoul, S. A. West, Kin selection for cooperation in natural bacterial populations. *Proc. Natl. Acad. Sci. U. S. A.* **119** (2022).
9. J. L. de Oliveira, *et al.*, Conditional expression explains molecular evolution of social genes in a microbe. *Nat. Commun.* **10**, 3284 (2019).
10. A. Ross-Gillespie, *et al.*, Frequency Dependence and Cooperation: Theory and a Test with Bacteria. *Am. Nat.* **170**, 331–342 (2007).
11. J. Gore, H. Youk, A. van Oudenaarden, Snowdrift game dynamics and facultative cheating in yeast. *Nature* **459**, 253–256 (2009).
12. P. G. Madgwick, L. J. Belcher, J. B. Wolf, Greenbeard Genes : Theory and Reality. *Trends Ecol. Evol.*, 1–12 (2019).
13. F. Tajima, Statistical method for testing the neutral mutation hypothesis by DNA polymorphism. *Genetics* (1989).
14. E. Paradis, Pegas: An R package for population genetics with an integrated-modular approach. *Bioinformatics* **26**, 419–420 (2010).
15. Y. X. Fu, W. H. Li, Statistical tests of neutrality of mutations. *Genetics* (1993) https:/doi.org/10.1093/genetics/133.3.693.
16. N. Stoletzki, A. Eyre-Walker, Estimation of the neutrality index. *Mol. Biol. Evol.* **28**, 63–70 (2011).
17. T. Zhou, W. Gu, C. O. Wilke, Detecting positive and purifying selection at synonymous sites in yeast and worm. *Mol. Biol. Evol.* **27**, 1912–1922 (2010).
18. D. Charif, J. R. Lobry, "SeqinR 1.0-2: a contributed package to the R project for statistical computing devoted to biological sequences retrieval and analysis." in *Structural Approaches to Sequence Evolution: Molecules, Networks, Populations*, U. Bastolla, M. Porto, H. Roman, M. Vendruscolo, Eds. (Springer Verlag, 2007) https:/doi.org/10.1007/978-3-540-35306-5_10.
19. V. Obenchain, *et al.*, VariantAnnotation: A Bioconductor package for exploration and annotation of genetic variants. *Bioinformatics* **30**, 2076–2078 (2014).
20. P. H. Brito, *et al.*, Genetic competence drives genome diversity in bacillus subtilis. *Genome Biol. Evol.* **10**, 108–124 (2018).
21. A. Dewar, *et al.*, Plasmids do not consistently stabilize cooperation across bacteria but may promote broad pathogen host-range. *Nat. Ecol. Evol.* **5**, 1624–1636 (2021).
22. T. W. Scott, S. A. West, A. E. Dewar, G. Wild, Is cooperation favoured by horizontal gene transfer? *GitHub, https://doi.org/10.5281/zenodo.7585371.* **7**, 113–120 (2023).
23. V. Molle, *et al.*, The Spo0A regulon of Bacillus subtilis. *Mol. Microbiol.* **50**, 1683–1701 (2003).
24. Y. Chai, F. Chu, R. Kolter, R. Losick, Bistability and biofilm formation in Bacillus subtilis. *Mol. Microbiol.* **67**, 254–263 (2008).
25. A. Dragoš, *et al.*, Division of Labor during Biofilm Matrix Production. *Curr. Biol.* **28**, 1903-1913.e5 (2018).
26. M. Kalamara, J. C. Abbott, C. E. Macphee, N. R. Stanley-Wall, Biofilm hydrophobicity in environmental isolates of Bacillus subtilis. *Microbiol. (United Kingdom)* **167** (2021).
27. T. A. Linksvayer, M. J. Wade, Genes with social effects are expected to harbor more sequence variation within and between species. *Evolution (N. Y).* **63**, 1685–1696 (2009).
28. E. P. C. Rocha, Neutral theory, microbial practice: Challenges in bacterial population genetics. *Mol. Biol. Evol.* **35**, 1338–1347 (2018).
29. A. Oslizlo, *et al.*, Exploring ComQXPA quorum-sensing diversity and biocontrol potential of Bacillus spp. isolates from tomato rhizoplane (2015)

https:/doi.org/10.1111/1751-7915.12258 (November 7, 2022).
30. H. T. Kiesewalter, *et al.*, Genomic and Chemical Diversity of Bacillus subtilis Secondary Metabolites against Plant Pathogenic Fungi. *mSystems* **6** (2021).
31. K. Steinke, O. S. Mohite, T. Weber, Á. T. Kovács, Phylogenetic Distribution of Secondary Metabolites in the Bacillus subtilis Species Complex. *mSystems* **6** (2021).
32. T. Pisithkul, *et al.*, Metabolic remodeling during biofilm development of bacillus subtilis. *MBio* **10** (2019).
33. F. M. Commichau, N. Pietack, J. Stülke, Essential genes in Bacillus subtilis: A re-evaluation after ten years. *Mol. Biosyst.* **9**, 1068–1075 (2013).
34. A. O. Urrutia, L. D. Hurst, The signature of selection mediated by expression on human genes. *Genome Res.* **13**, 2260–2264 (2003).
35. S. Arnaouteli, N. C. Bamford, N. R. Stanley-Wall, Á. T. Kovács, Bacillus subtilis biofilm formation and social interactions. *Nat. Rev. Microbiol.* **19**, 600–614 (2021).
36. M. Kalamara, M. Spacapan, I. Mandic-Mulec, N. R. Stanley-Wall, Social behaviours by Bacillus subtilis: quorum sensing, kin discrimination and beyond. *Mol. Microbiol.* **110**, 863–878 (2018).
37. R. J. Morris, *et al.*, Natural variations in the biofilm-associated protein BslA from the genus Bacillus. *Sci. Rep.* **7** (2017).
38. M. Miethke, *et al.*, Ferri-bacillibactin uptake and hydrolysis in Bacillus subtilis. *Mol. Microbiol.* **61**, 1413–1427 (2006).
39. H. Pi, J. D. Helmann, Sequential induction of Fur-regulated genes in response to iron limitation in Bacillus subtilis. *Proc. Natl. Acad. Sci. U. S. A.* **114**, 12785–12790 (2017).
40. T. Bucher, *et al.*, An active β-lactamase is a part of an orchestrated cell wall stress resistance network of Bacillus subtilis and related rhizosphere species. *Environ. Microbiol.* **21**, 1068–1085 (2019).
41. N. Noguchi, M. Sasatsu, M. Kono, "Genetic mapping in Bacillus subtilis 168 of the aadK gene which encodes arninoglycoside 6-adenylyltransferase" (1993).
42. C. Torres, C. Galián, C. Freiberg, J. R. Fantino, J. M. Jault, The YheI/YheH heterodimer from Bacillus subtilis is a multidrug ABC transporter. *Biochim. Biophys. Acta* **1788**, 615–622 (2009).
43. O. Ertekin, *et al.*, Analysis of a bac operon-silenced strain suggests pleiotropic effects of bacilysin in Bacillus subtilis. *J. Microbiol.* **58**, 297–313 (2020).
44. K. Kobayashi, Diverse LXG toxin and antitoxin systems specifically mediate intraspecies competition in Bacillus subtilis biofilms. *PLOS Genet.* **17**, e1009682 (2021).
45. C. R. Harwood, Y. Kikuchi, The ins and outs of Bacillus proteases: Activities, functions and commercial significance. *FEMS Microbiol. Rev.* **46**, 1–20 (2022).
46. D. Romero, *et al.*, The Iturin and Fengycin Families of Lipopeptides Are Key Factors in Antagonism of Bacillus subtilis Toward Podosphaera fusca. *MPMI* **20**, 430–440 (2007).
47. S. Müller, *et al.*, Bacillaene and Sporulation Protect Bacillus subtilis from Predation by Myxococcus xanthus. *Appl. Environ. Microbiol.* **80**, 5603 (2014).
